# Differences in Intracellular Fate of Two Spotted Fever Group *Rickettsia* in Macrophage-Like Cells

**DOI:** 10.3389/fcimb.2016.00080

**Published:** 2016-07-29

**Authors:** Pedro Curto, Isaura Simões, Sean P. Riley, Juan J. Martinez

**Affiliations:** ^1^PhD Programme in Experimental Biology and Biomedicine, Center for Neuroscience and Cell Biology, University of CoimbraCoimbra, Portugal; ^2^Institute for Interdisciplinary Research, University of CoimbraCoimbra, Portugal; ^3^Center for Neuroscience and Cell BiologyCoimbra, Portugal; ^4^Vector Borne Disease Laboratories, Department of Pathobiological Sciences, LSU School of Veterinary MedicineBaton Rouge, LA, USA; ^5^Biocant, Biotechnology Innovation CenterCantanhede, Portugal

**Keywords:** rickettsiae, spotted fever group *Rickettsia*, macrophages, pathogenicity, intracellular fate, *R. conorii*, *R. montanensis*

## Abstract

Spotted fever group (SFG) rickettsiae are recognized as important agents of human tick-borne diseases worldwide, such as Mediterranean spotted fever (*Rickettsia conorii*) and Rocky Mountain spotted fever (*Rickettsia rickettsii*). Recent studies in several animal models have provided evidence of non-endothelial parasitism by pathogenic SFG *Rickettsia* species, suggesting that the interaction of rickettsiae with cells other than the endothelium may play an important role in pathogenesis of rickettsial diseases. These studies raise the hypothesis that the role of macrophages in rickettsial pathogenesis may have been underappreciated. Herein, we evaluated the ability of two SFG rickettsial species, *R. conorii* (a recognized human pathogen) and *Rickettsia montanensis* (a non-virulent member of SFG) to proliferate in THP-1 macrophage-like cells, or within non-phagocytic cell lines. Our results demonstrate that *R. conorii* was able to survive and proliferate in both phagocytic and epithelial cells *in vitro*. In contrast, *R. montanensis* was able to grow in non-phagocytic cells, but was drastically compromised in the ability to proliferate within both undifferentiated and PMA-differentiated THP-1 cells. Interestingly, association assays revealed that *R. montanensis* was defective in binding to THP-1-derived macrophages; however, the invasion of the bacteria that are able to adhere did not appear to be affected. We have also demonstrated that *R. montanensis* which entered into THP-1-derived macrophages were rapidly destroyed and partially co-localized with LAMP-2 and cathepsin D, two markers of lysosomal compartments. In contrast, *R. conorii* was present as intact bacteria and free in the cytoplasm in both cell types. These findings suggest that a phenotypic difference between a non-pathogenic and a pathogenic SFG member lies in their respective ability to proliferate in macrophage-like cells, and may provide an explanation as to why certain SFG rickettsial species are not associated with disease in mammals.

## Introduction

Rickettsiae are small Gram-negative, obligate intracellular α-proteobacteria transmitted to humans through arthropod vectors (Hackstadt, 1996). The rapid increase in *Rickettsia* genome sequences allowed their classification into several distinct genetic groups including the ancestral group (AG), spotted fever group (SFG), typhus group (TG), and transitional group (TRG; Gillespie et al., 2008; Fournier and Raoult, 2009; Goddard, 2009; Weinert et al., 2009). Many rickettsial species belonging to the TG and SFG are pathogenic to humans, causing serious illness such as epidemic typhus (*Rickettsia prowazekii*), Rocky Mountain spotted fever (RMSF; *Rickettsia rickettsii*), and Mediterranean spotted fever (MSF; *Rickettsia conorii*; Parola et al., 2005; Walker, 2007; Walker and Ismail, 2008). However, it has been reported that members of each group can drastically differ in their ability to cause disease (Uchiyama, 2012; Wood and Artsob, 2012). The SFG *Rickettsia* species, *R. montanensis*, has been detected in *Dermacentor variabilis* ticks throughout the United States and Canada, but is considered an organism with limited or no pathogenicity to humans (Ammerman et al., 2004; Carmichael and Fuerst, 2010; McQuiston et al., 2012). A previous report has demonstrated that prior exposure to *R. montanensis* may confer protective immunity to mammalian hosts that are subsequently infected by *R. rickettsii*, possibly by preventing these mammals from becoming amplifying hosts for virulent rickettsial species (Moncayo et al., 2010). Conversely, *R. conorii* the causative agent of MSF (considered as a highly pathogenic organism) is associated with morbidity, and fatality rates varying from 21 to 33% in Portugal (Walker, 1989; de Sousa et al., 2003; Galvao et al., 2005). MSF is endemic to Southern Europe, North Africa, and India (Rovery et al., 2008); however, recent evidence has unveiled that MSF exhibits an expansive geographic distribution, now including central Europe and central and southern Africa (Wood and Artsob, 2012).

Although the progression of rickettsial diseases in humans has been the subject of several studies over the last years, the underlying mechanisms that are responsible for differences in pathogenicity by different rickettsiae species are still to be understood. The establishment of a successful infection by a pathogen involves the recognition and invasion of target cells in the host, adaptation to the intracellular environment, replication, and ultimately dissemination within the host (Walker and Ismail, 2008). Although endothelial cells have long been considered the main target cells for rickettsiae, infection of monocytes/macrophages and hepatocytes has also been previously reported (Walker and Gear, 1985; Walker et al., 1994, 1997, 1999). Additionally, mouse and Rhesus macaque models of SFG *Rickettsia* infection have provided evidence of non-endothelial parasitism by *R. conorii* and *R. parkeri*, respectively (Banajee et al., 2015; Riley et al., 2016). Using C3H/HeN mice as a fatal murine model of MSF, Riley et al. have demonstrated evidence of numerous bacteria within the cytoplasm of macrophages and neutrophils, both in tissues and within the blood circulation. In the Rhesus macaque model, *R. parkeri* was present at cutaneous inoculation sites, primarily within macrophages and occasionally neutrophils. These results suggest that the interaction of rickettsiae with cells other than the endothelium may play an important role in the pathogenesis of rickettsial diseases, and is an underappreciated aspect of rickettsial biology. There are a few reports studying the interaction of different rickettsial species with macrophages *in vitro* (Gambrill and Wisseman, 1973a,b; Feng and Walker, 2000); however, the role of macrophages in rickettsial pathogenesis remains to be clarified. Therefore, more studies are required to better understand the biological function of macrophages during rickettsial infections.

In this work, we report that *R. conorii*, a pathogenic member of SFG rickettsiae, is able to invade and proliferate within THP-1-derived macrophages, whereas *R. montanensis*, a non-pathogenic member of SFG *Rickettsia*, is drastically compromised in the ability to proliferate within these cells. These findings suggest that the intracellular fate in macrophages may provide an explanation as to why certain SFG rickettsial species are not associated with disease.

## Materials and methods

### Cell lines, *Rickettsia* growth and purification

Vero and EA.hy926 cells were grown in Dulbecco's modified Eagle's medium (DMEM; Gibco) supplemented with 10% heat-inactivated fetal bovine serum (Atlanta Biologicals), 1x non-essential amino acids (Corning), and 0.5 mM sodium pyruvate (Corning). THP-1 (ATCC TIB-202™) cells were grown in RPMI-1640 medium (Gibco) supplemented with 10% heat-inactivated fetal bovine serum. Differentiation of THP-1 cells into macrophage-like cells was carried out by the addition of 100 nM of phorbol 12-myristate 13-acetate (PMA; Fisher). Cells were allowed to differentiate and adhere for 3 days prior to infection. All cell lines were maintained in a humidified 5% CO_2_ incubator at 34°C. *R. conorii* isolate Malish7 and *R. montanensis* isolate M5/6 were propagated in Vero cells and purified as previously described (Ammerman et al., 2008; Chan et al., 2009, 2011).

### Antibodies

Anti-Rc_PFA_, rabbit polyclonal antibody that recognizes *R. conorii*, was generated as previously described (Chan et al., 2011; Cardwell and Martinez, 2012). Anti-*Rickettsia* rabbit polyclonal antibody that recognizes *R. montanensis* (NIH/RML I7198) was kindly provided by Dr. Ted Hackstadt (Rocky Mountain Laboratories). Alexa Fluor 488- and 546-conjugated goat anti-rabbit IgG, Texas Red-X-phalloidin, and DAPI (4′, 6′-diamidino-2-phenylindole) were purchased from Thermo Scientific. Anti-LAMP2 [H4B4] and anti-cathepsin D [CTD19] antibodies were purchased from abcam.

### Assessment of *Rickettsia* growth dynamics

Growth curves were performed by inoculating *R. conorii* and *R. montanensis* at a multiplicity of infection (MOI) of 2.5 into Vero, EA.hy926, or PMA-differentiated THP-1 cells monolayers at a confluency of 2 × 10^5^ cells per well, in 24 well plates, with 3 wells infected for each day of the growth curve. Plates were centrifuged at 300 × g for 5 min at room temperature to induce contact between rickettsiae and host cells, and incubated at 34°C and 5% CO_2_. At each specific time point post inoculation, cells were scraped and samples were stored in PBS at −80°C. For undifferentiated THP-1 cells, 2 × 10^5^ cells were infected with *R. conorii* and *R. montanensis* at a MOI of 2.5 in a total volume of 100 μL. Samples were centrifuged at 300 × g for 5 min at room temperature to induce contact between rickettsiae and host cells, and then transferred to 96 well plates and incubated at 34°C and 5% CO_2_ (3 samples infected for each day of the growth curve). At each specific time point post inoculation, samples were stored in PBS at −80°C. Genomic DNA was extracted using the PureLink Genomic DNA kit (Life Technologies) according to the manufacturer's instructions. The extracted DNA was subjected to quantitative PCR analysis using LightCylcer 480 II (Roche). Bacterial growth was queried by quantitative PCR using TaqMan Master Mix at 95°C, with a 10 min incubation followed by 50 cycles of 95°C 15 s and 58°C 1 min. The rickettsial *sca1* gene was amplified using the primers sca1-F, sca1-R, and Sca1-Fam and the mammalian *actin* gene was amplified using the primers actin-F, actin-R, and actin-Hex (Vic; Supplementary Table 1). Growth is presented as the ratio of *sca1* vs. *actin*. All unknowns were quantified by ΔΔCt as compared to molar standards. Experiments were done in triplicate with duplicates for each experiment.

Growth dynamics were also assessed by immunofluorescence. Briefly, PMA-differentiated THP-1, Vero, and EA.hy926 cells were seeded onto glass coverslips in 24-well plates at 2 × 10^5^ cells per well. Infections were performed as described above. At each indicated time point post inoculation, infected monolayers were washed with PBS and fixed in 4% paraformaldehyde (PFA) for 20 min. For undifferentiated THP-1 cells, the cells were harvested, washed with PBS, attached to slides by centrifugation (800 rpm, 8 min), and cells were fixed in 4% paraformaldehyde (PFA) for 20 min. All samples were then permeabilized with 0.1% Triton X-100 and blocked with 2% BSA. *R. conorii* growth dynamics were assessed by staining with anti-Rc_PFA_ (1:1000) followed by Alexa Fluor 488-conjugated goat anti-rabbit IgG (1:1000), DAPI (1:1000), and Texas Red-X-phalloidin (1:200). For *R. montanensis*, staining was carried out with NIH/RML I7198 (1:1500) followed by Alexa Fluor 488-conjugated goat anti-rabbit IgG (1:1000), DAPI (1:1000) and Texas Red-X-phalloidin (1:200). After washing with PBS, glass coverslips were mounted in Mowiol mounting medium and preparations were viewed on a LEICA DM 4000 B microscope equipped with Nuance FX multispectral imaging system using a final X100 optical zoom and processed with Image J software.

### Electron microscopy

For transmission electron microscopy (TEM), 12 wells of PMA-differentiated THP-1 cells in 6 well plates were inoculated with *R. conorii* (MOI = 2.5). After 5 days in culture, cells were scraped, centrifuged at 10,000 × g for 7 min at room temperature and washed with PBS. After this washing step, cells were centrifuged under the same conditions, fixed in primary fixative solution (1.6% paraformaldehyde, 2.5% glutaraldehyde, 0.03% CaCl_2_ in 0.05M cacodylate buffer, pH 7.4), pelleted, and embedded in 3% agarose. Agar blocks were cut in 1 mm^3^ cubes and transferred to a fresh portion of the fixative for 2 h at room temperature. Samples were then washed in 0.1 M cacodylate buffer supplemented with 5% sucrose, postfixed in 1% osmium tetroxide for 1 h, washed in water, and in-block stained with 2% uranyl acetate in 0.2 M sodium acetate buffer, pH 3.5. Specimens were dehydrated in ascending ethanol series and propylene oxide, and embedded in Epon-Araldite mixture. Blocks were sectioned with the Ultratome Leica EM UC7. Thin (80 nm) sections were stained with lead citrate for 5 min and examined in JEOL JEM 1011 microscope with the attached HAMAMATSU ORCA-HR digital camera. All reagents for electron microscopy were from EMS (Hatfield, PA).

### Cell association and invasion assays

Cell association and invasion assays were performed as previously described with some modifications (Martinez and Cossart, 2004). Briefly, mammalian cells (THP-1 and Vero) were seeded on glass coverslips in 24-well plates at 2 × 10^5^ cells per well. PMA-differentiated THP-1 and Vero cells were infected with *R. conorii* and *R. montanensis* (MOI = 10), the plates were centrifuged at 300 × g for 5 min at room temperature to induce contact, and subsequently incubated for 60 min at 34°C and 5% CO_2_. Infected monolayers were washed 1x with 1 mL PBS, and fixed in 4% PFA for 20 min prior to staining. For cell association assays, after permeabilization with 0.1% Triton X-100 and blocking with 2% BSA, *R. conorii* were stained with anti-Rc_PFA_ (1:1000) followed by Alexa Fluor 488-conjugated goat anti-rabbit IgG, DAPI (1:1000) and Texas Red-X-phalloidin (1:200). For *R. montanensis*, staining was carried out with NIH/RML I7198 antibody (1:1500) followed by Alexa Fluor 488-conjugated goat anti-rabbit IgG, DAPI (1:1000) and Texas Red-X-phalloidin (1:200). Experiments were done in triplicate and results of each experiment were expressed as the ratio of rickettsiae cells to mammalian cells (nuclei). At least 200 nuclei were counted for each experiment. For invasion assays, infected monolayers were processed for differential staining to distinguish between extracellular and intracellular rickettsia. Briefly, extracellular *R. conorii* were stained with anti-Rc_PFA_ (1:1000) followed by Alexa Fluor 546-conjugated goat anti-rabbit IgG (1:1000), prior to permeabilization of the mammalian cells with 0.1% Triton X-100. After permeabilization, the total *R. conorii* cells were then stained with anti-Rc_PFA_ (1:1000) followed by Alexa Fluor 488-conjugated goat anti-rabbit IgG (1:1000). Invasion assays of *R. montanensis* were assessed using the same procedure, and *R. montanensis* staining was carried out with NIH/RML I7198 antibody (1:1500). Bacteria staining positive for Alexa Fluor 546-conjugated goat anti-rabbit IgG were considered as external while bacteria stained for both secondary antibodies were considered as total bacteria present. The number of internalized rickettsiae was determined by the difference between total and external rickettsiae, and results are expressed as percentages of internalized rickettsiae. As for association assays, experiments were done in triplicate with at least 200 nuclei for each experiment. Images were digitally captured with an OLYMPUS IX71 inverted microscope (Tokyo, Japan) equipped with an OLYMPUS DP72 camera (Tokyo, Japan) using a final X40 optical zoom. Rickettsiae and mammalian nuclei were counted using the cell counter analysis tool from ImageJ (http://rsb.info.nih.gov/ij). Statistical analysis was performed by unequal variance *t*-test (Welch's *t*-test) using Prism software package (GraphPad Software Inc.).

### LAMP-2 and cathepsin D immunostaining and confocal microscopy

Mammalian cells (THP-1 and Vero) were seeded into 24-well plates under coverslips for a cell confluency of 2 × 10^5^ cells per well. PMA-differentiated THP-1 and Vero cells were infected with *R. conorii* and *R. montanensis* (MOI = 10), the plates centrifuged at 300 × g for 5 min at room temperature to induce contact, and subsequently incubated for 60 min or 24 h at 34°C and 5% CO_2_. Infected monolayers were washed with PBS, and fixed in 4% paraformaldehyde (PFA) for 20 min prior to staining. After permeabilization, the cells were incubated with primary antibodies anti-Rc_PFA_ (1:1000; *R. conorii*); NIH/RML I7198 antibody (1:1500; *R. montanensis)*, and mouse anti-LAMP-2 (1:100) or anti-cathepsin D (1:5500; lysosome markers), followed by Alexa Fluor 546-conjugated goat anti-rabbit IgG (1:1000) and Alexa Fluor 488-conjugated goat anti-mouse (IgG; 1:1000). Images were acquired using a confocal laser scanning microscope Leica TCS SP2 microscope with a × 100 oil immersion objective and processed using ImageJ software. Analysis of fluorescence intensity was performed with the RGB profiler plugin within the ImageJ software package (https://imagej.nih.gov/ij/).

## Results

### *R. conorii* is able to invade and grow inside macrophage-like cells

**Figure 1:**
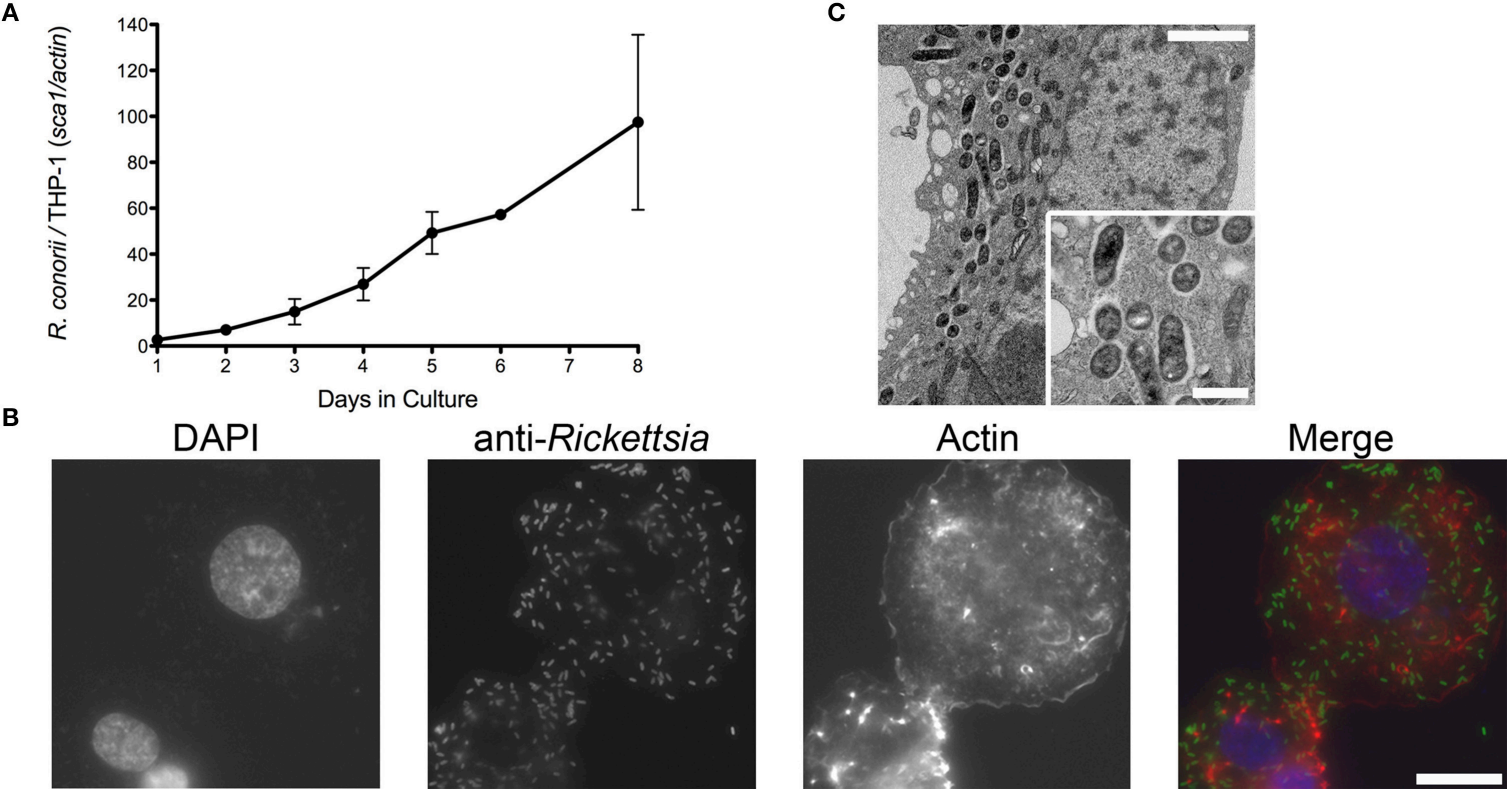
**Ability of ***R. conorii*** to invade and proliferate within THP-1-derived macrophages. (A)** PMA-differentiated THP-1 cells were infected with *R. conorii* and genomic DNA was extracted at different time-points after infection. Quantitative PCR data are expressed as the ratio of *R. conorii sca1* vs. *actin* DNA content. **(B)** Immunofluorescence microscopy of THP-1-derived macrophages cells infected with *R. conorii* at 3 days post-infection. Cells were stained with DAPI (blue) to identify host nuclei, Phalloidin (red) to stain actin and anti-*Rickettsia* antibody (RcPFA) followed by Alexa Fluor 488 (green) to identify *R. conorii*. Scale bar = 10 μm. **(C)** Ultrastructure of THP-1-derived macrophages after 5 days post inoculation with *R. conorii* by TEM. Scale bar = 2 μm (top) and 500 nm (bottom).

Infection of endothelial cells by SFG rickettsiae has been previously reported by several groups (Walker et al., 1994; Walker, 1997; Bechah et al., 2008b; Colonne et al., 2011). In addition, evidence of non-endothelial parasitism of *R. conorii in vivo* has also been recently reported, suggesting that the interaction with cells other than endothelial cells could be relevant to rickettsial pathogenesis (Riley et al., 2016). To further evaluate the growth dynamics of *R. conorii* in macrophage-like cells, human THP-1 monocytes were differentiated into macrophages by incubation with PMA, and infected with *R. conorii* at a MOI of 2.5. Samples were collected from these cultures at several time-points post inoculation, and total genomic DNA was extracted. As illustrated in Figure 1A, q-PCR analysis of the ratio of *R. conorii* (*sca1*) to THP-1 (*actin*) DNA content clearly demonstrated that *R. conorii* was able to grow in PMA-differentiated THP-1 cultures. This successful ability of *R. conorii* to proliferate in THP-1-derived macrophages was also confirmed by immunofluorescence microscopy of cells 3 days post inoculation, with the clear presence of anti-Rc_PFA_-positive intact bacteria dispersed within the mammalian cells (Figure 1B). To evaluate in more detail the morphology of *R. conorii* in THP-1-derived macrophages, TEM was carried out. At day 5 post inoculation, TEM images confirmed the presence of intact bacteria spread throughout the cytoplasm of the cells (Figure 1C). Interestingly, most of these bacteria displayed a normal morphology, and were not surrounded by membranes or phagolysosome-like structures but free in the cytoplasm, with an electron-lucent zone adjacent to the bacterial membrane. These results clearly indicate that *R. conorii* is able to survive and proliferate in the hostile environment of THP-1-derived macrophages.

### *R. montanensis* is able to grow in non-phagocytic mammalian cells but not in human macrophage-like cells

**Figure 2:**
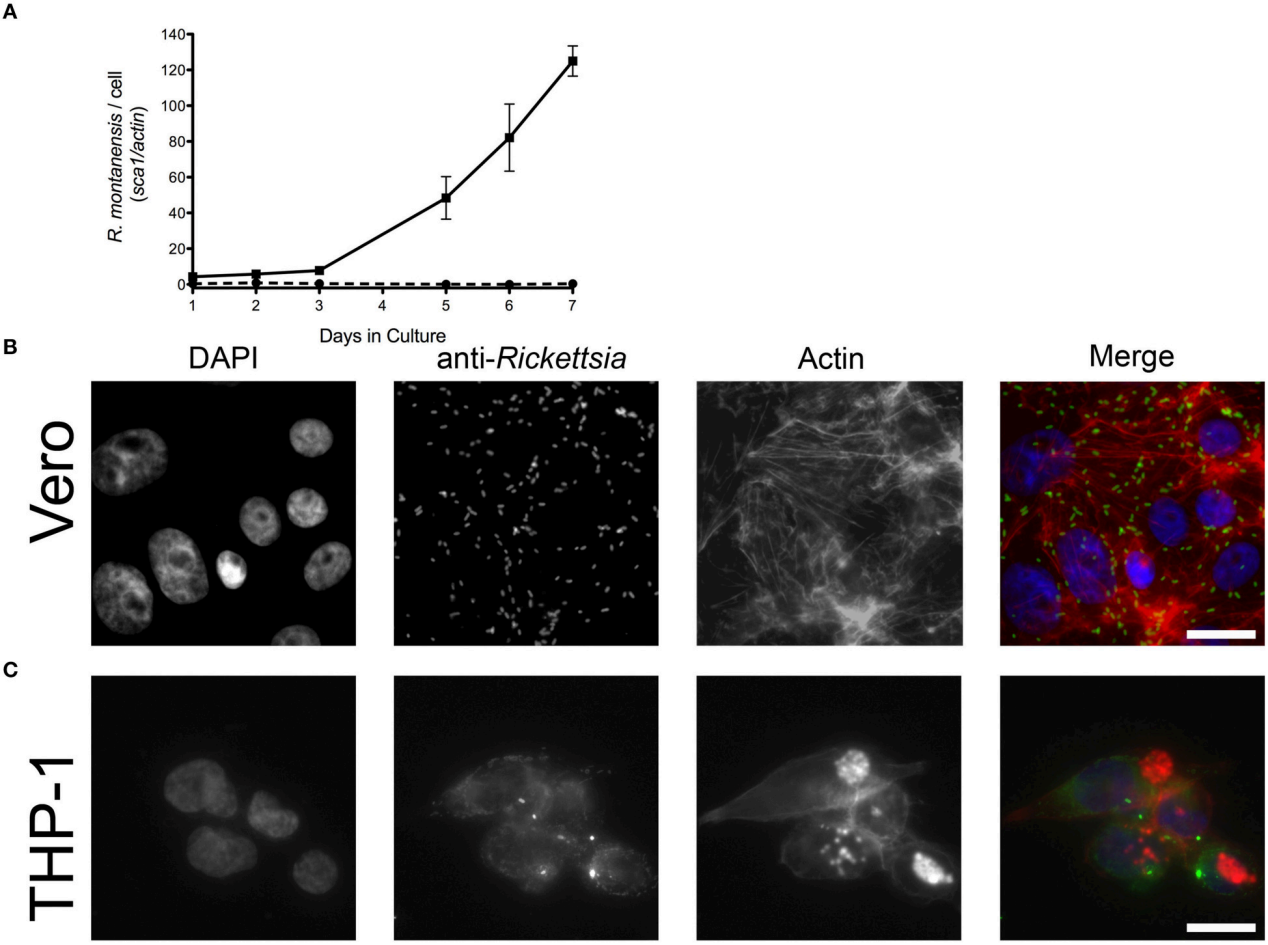
*****R. montanensis*** is able to grow inside epithelial cells (Vero) but not in THP-1 derived macrophages. (A)** PMA-differentiated THP-1 cells (dashed lines) and Vero cells (solid lines) were infected with *R. montanensis*, and genomic DNA was extracted at different time-points after infection. Quantitative PCR data are expressed as the ratio of *R. montanensis sca1* vs. *actin* DNA content. **(B**,**C)** Immunofluorescence microscopy of Vero cells **(B)** and THP-1-derived macrophages **(C)** infected with *R. montanensis* at 3 days after infection. Cells were stained with DAPI (blue) to stain host nuclei, Phalloidin (red) to stain actin and rabbit anti-*Rickettsia* polyclonal antibody NIH/RML I7198 followed by Alexa Fluor 488 (green) to stain *R. montanensis*. Scale bar = 10 μm.

*R. montanensis* has traditionally been considered a nonpathogenic member of the SFG rickettsiae, and only a limited number of human infections have been previously reported with this organism (McQuiston et al., 2012). We sought to determine if *R. montanensis* would behave similarly to *R. conorii* and proliferate within epithelial and macrophage-like cells. Both THP-1-derived macrophages and Vero cells were infected with *R. montanensis* at a MOI of 2.5, and samples were collected from these cultures at several time-points post-inoculation for q-PCR analysis. As previously described, the ratio of *R. montanensis* (*sca1*) to mammalian cell (*actin*) DNA content was used to evaluate the growth dynamics of *R. montanensis* in both cell lines over time. As shown in Figure 2A, *R. montanensis* was able to grow in Vero cells, but was not able to proliferate in THP-1-derived macrophages. These results were also confirmed by immunofluorescence microscopy (Figures 2B,C). Moreover, *R. montanensis* was able to invade and proliferate in the cultured human endothelial cell line, EA.hy926 (Supplementary Figure 1). Fluorescent microscopy analysis of Vero cells infected with *R. montanensis* after 3 days post inoculation revealed intact bacilli dispersed within the host cytoplasm (Figure 2B); however, few intact bacteria were found after 3 days of inoculation of *R. montanensis* in THP-1-derived macrophages (Figure 2C). A similar phenotype was also observed when undifferentiated THP-1 cells were infected (Supplementary Figure 2). These data demonstrate that there is a difference in the ability of *R. montanensis* to proliferate within both undifferentiated THP-1 cells (monocytic) and THP-1 derived macrophages when compared with other cell types, in contrast to the observed growth of *R. conorii*.

### Binding of *R. montanensis* to THP-1-derived macrophages is compromised but they still can invade

**Figure 3:**
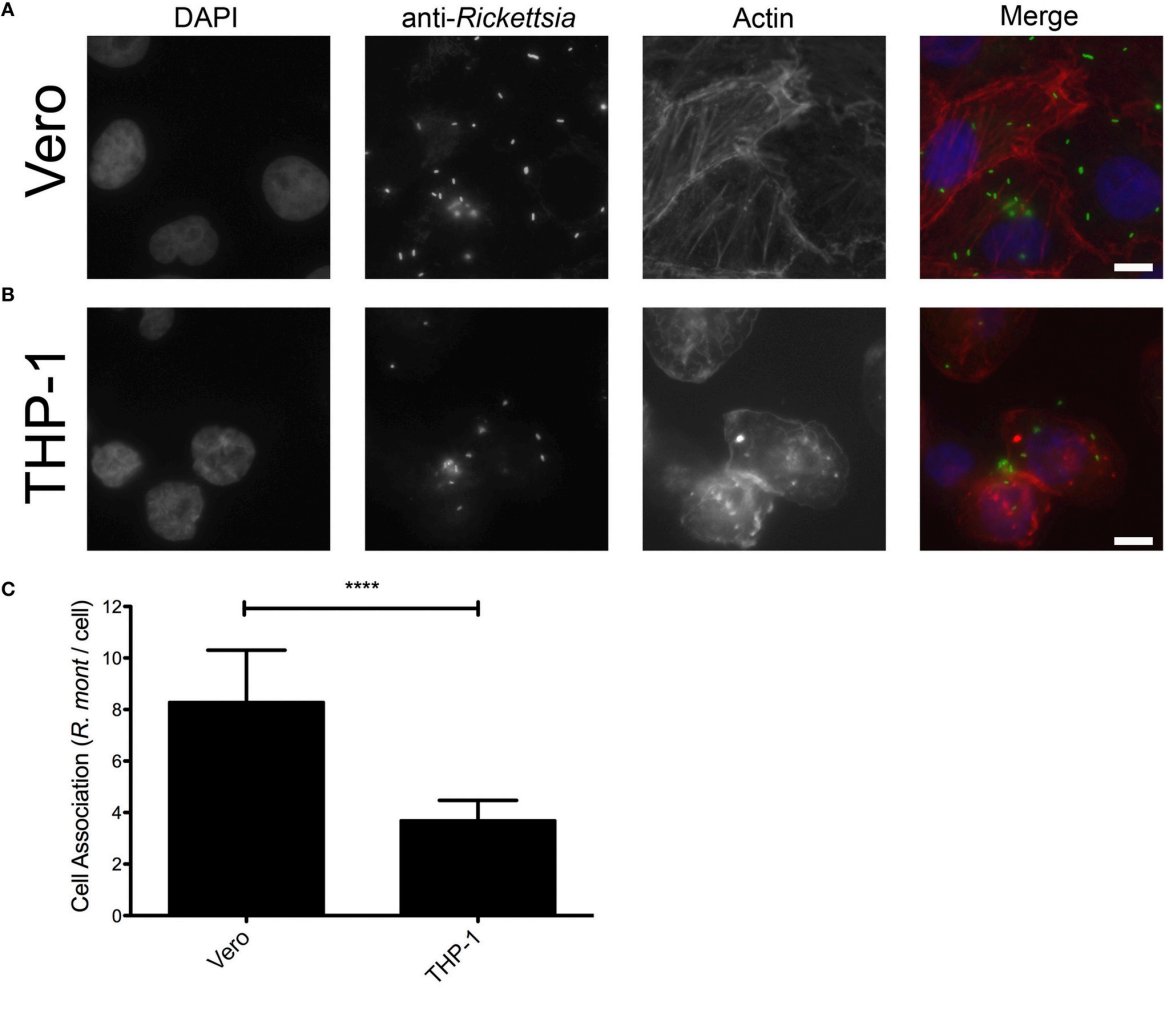
*****R. montanensis*** shows a defect in association with THP-1-derived macrophages**. PMA-differentiated THP-1 cells and Vero cells were infected with *R. montanensis* (MOI = 10). After 60 min of infection, cells were fixed and stained for immunofluorescence analysis with rabbit anti-*Rickettsia* polyclonal antibody (NIH/RML I7198), followed by Alexa Fluor 488 (green) to stain *R. montanensis*, DAPI to visualize the host nuclei (blue) and Phalloidin to illustrate the host cytoplasm (red). **(A**,**B)** Representative immunofluorescence images of *R. montanensis* association assays in Vero **(A)** and macrophage-like **(B)** cells. Each row shows, from left to right nuclei staining, rickettsia staining, actin staining, and the merged image. Scale bar = 10 μm. **(C)** *Rickettsia* and mammalian cells were counted and results are expressed as the ratio of rickettsiae to mammalian cells. At least 200 host nuclei were counted for each experimental condition. Results are shown as the mean ± SD (^****^P < 0.0001).

Adherence and subsequent invasion to the target cells is a critical step in the establishment of a successful rickettsial infection (Martinez and Cossart, 2004). We hypothesized that *R. montanensis* may be unable to adhere to and subsequently invade into THP-1-derived macrophages. To test this, we initially analyzed the adherence capacity of *R. montanensis* in both cell types. Vero and THP-1 cells were inoculated with *R. montanensis* (MOI = 10) for 60 min, and the ability to associate with cultured mammalian cells *in vitro* was assessed by immunofluorescence and quantification of the ratio of *Rickettsia* cells per mammalian cell nucleus. As shown in Figure 3, the ability of *R. montanensis* to bind to THP-1-derived macrophages was significantly decreased compared to the binding to Vero cells. Representative immunofluorescence microscopy images (Figures 3A,B) confirmed these differences. As a control, association assays with *R. conorii* were also performed in both cell types. Our results suggest that adherence of *R. conorii* to THP-1-derived macrophages was not compromised (Supplementary Figure 3). Together, these data suggest that *R. montanensis* are defective in binding to THP-1-derived macrophages when compared with their capacity to bind to Vero cells.

**Figure 4:**
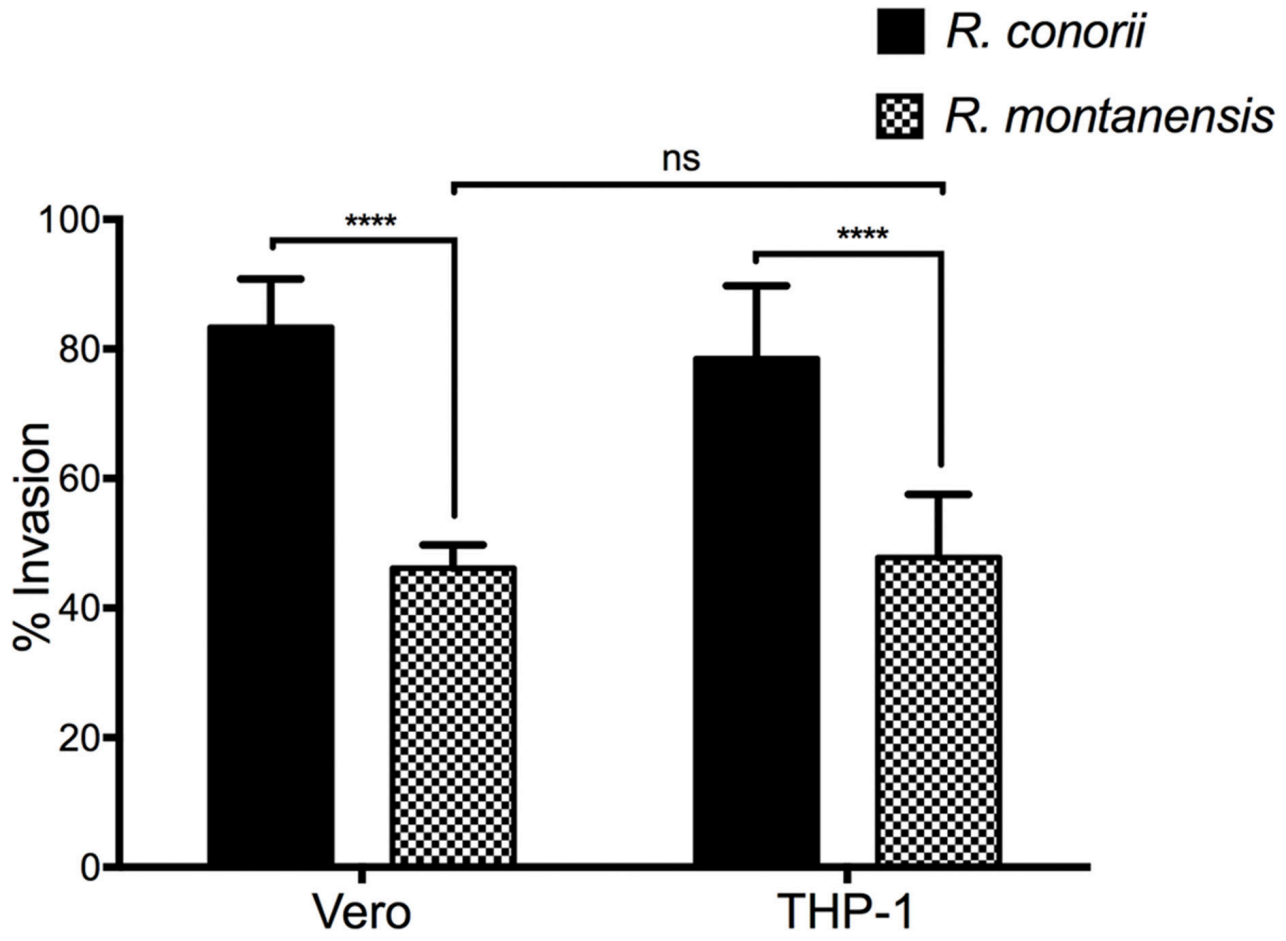
**Invasion rate of ***R. montanensis*** into THP-1-derived macrophages is not affected when compared with Vero cells**. PMA-differentiated THP-1 cells and Vero cells were infected with *R. montanensis* and *R. conorii* (MOI = 10). After 60 min of infection, cells were fixed and processed for differential staining to distinguish between extracellular and intracellular rickettsiae. Results are expressed as percentage of internalized rickettsiae. At least 200 host nuclei were counted for each experimental condition. Results are shown as the mean ± SD (ns, non-significant, ^****^P < 0.0001).

We next sought to determine whether the remaining *R. montanensis* cells bound to THP-1 cells were still capable of inducing their internalization into these phagocytic cells. To address this, we performed invasion assays of *R. montanensis* in Vero cells and THP-1-derived macrophages. Similar assays using *R. conorii* were performed as a control. Both species (MOI = 10) were used to inoculate each cell-type for 60 min. Samples were processed for differential staining to distinguish between extracellular and intracellular rickettsiae that were then quantified to determine the percentage of internalized bacteria. As shown in Figure 4, the invasion rate of *R. montanensis* into THP-1-derived macrophages was not significantly affected when compared with that observed in Vero cells. Although the ability of *R. montanensis* to bind to THP-1-derived macrophages was significantly decreased in the association assays, these results suggest that those bacteria that bind are still able to invade these cells.

### *Rickettsia montanensis* is rapidly destroyed in THP-1-derived macrophages

**Figure 5:**
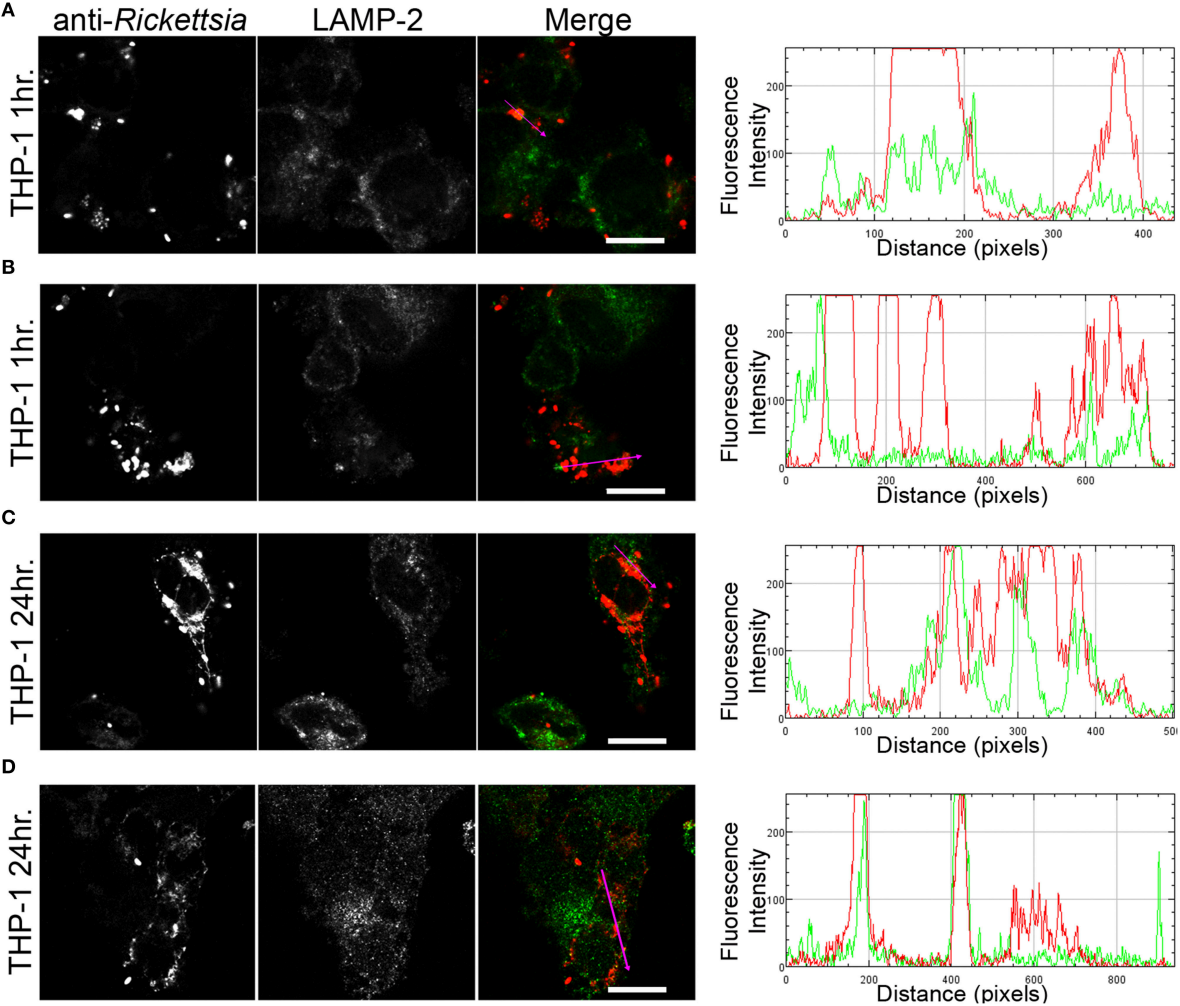
*****R. montanensis*** is rapidly destroyed in THP-1-derived macrophages**. THP-1-derived macrophages were infected with *R. montanensis* (MOI = 10). At 60 min or 24 h post infection, cells were fixed, permeabilized, and double stained for immunofluorescence confocal microscopy analysis with NIH/RML I7198 followed by Alexa Fluor 546 (red) to stain *R. montanensis*, and the monoclonal antibody for LAMP-2, lysosomal membrane protein followed by Alexa Fluor 488 (green). **(A–D)** Representative images of a single slice from the z stacks. THP1-derived macrophages at 60 min post infection **(A,B)** and 24 h post infection **(C,D)**. Each row shows, from left to right, *Rickettsia* staining, LAMP-2 staining, the merged image, and a RGB plot profile illustrating the fluorescence intensity along the magenta arrow. Scale bar = 10 μm. Supplementary Movies 1, 2 represent 360° rotation movie of the 3D projection of the stack images shown in **(A)** and **(C)**, respectively.

**Figure 6:**
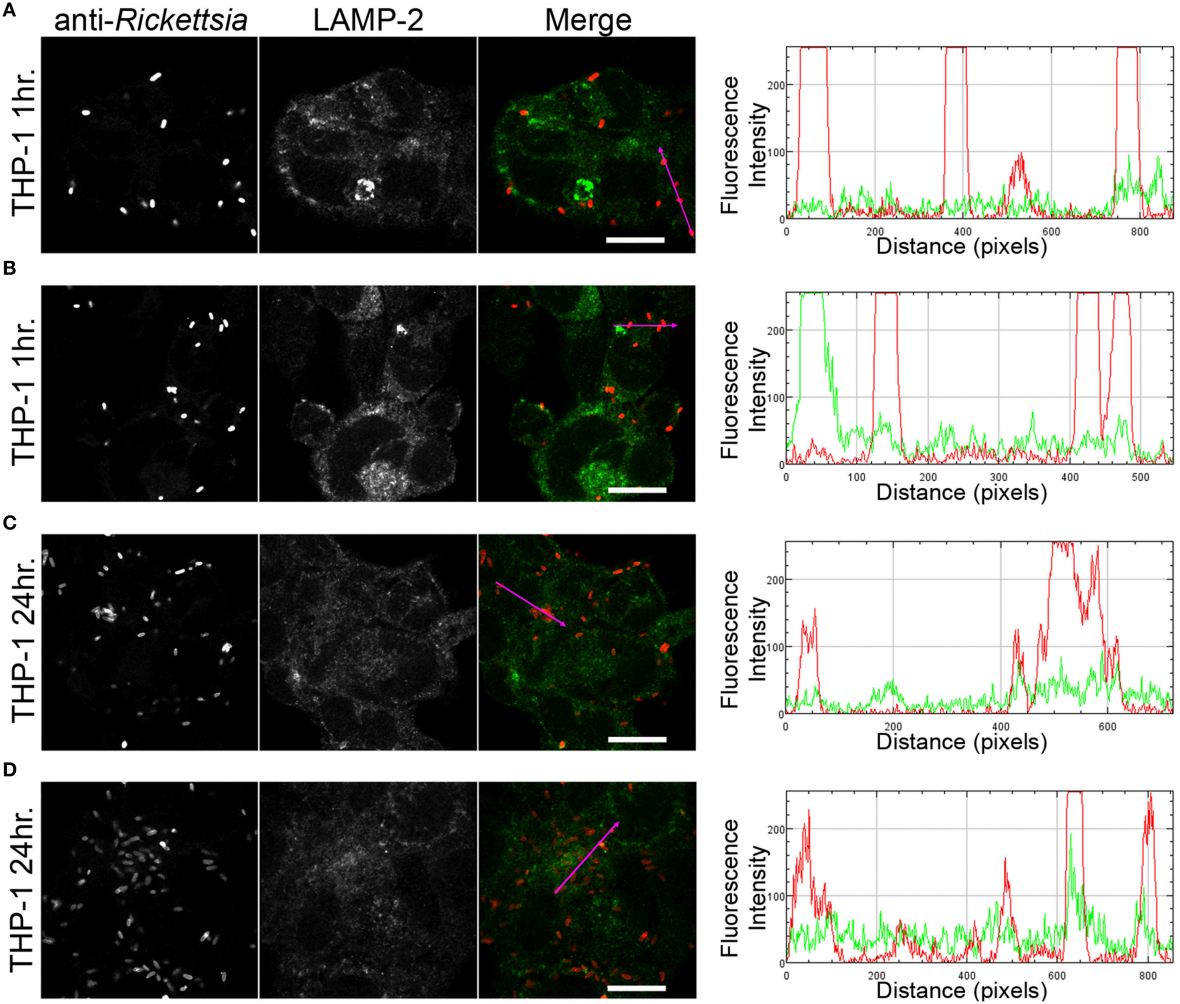
*****R. conorii*** is maintained as morphologically intact bacteria in THP-1-derived macrophages**. THP-1-derived macrophages were infected with *R. conorii* (MOI = 10). At 60 min or 24 h post infection, cells were fixed, permeabilized, and double stained for immunofluorescence confocal microscopy analysis with anti-Rc_PFA_ followed by Alexa Fluor 546 (red) to stain *R. conorii* and the monoclonal antibody for LAMP-2, lysosomal membrane protein followed by Alexa Fluor 488 (green). **(A–D)** Representative images of a single slice from the z stacks. THP1-derived macrophages at 60 min post infection **(A,B)** and 24 h post infection **(C,D)**. Each row shows, from left to right, *Rickettsia* staining, LAMP-2 staining, the merged image, and a RGB plot profile illustrating the fluorescence intensity along the magenta arrow. Scale bar = 10 μm. Supplementary Movies 5, 6 represent 360° rotation movie of the 3D projection of the stack images shown in **(A)** and **(C)**, respectively.

**Figure 7:**
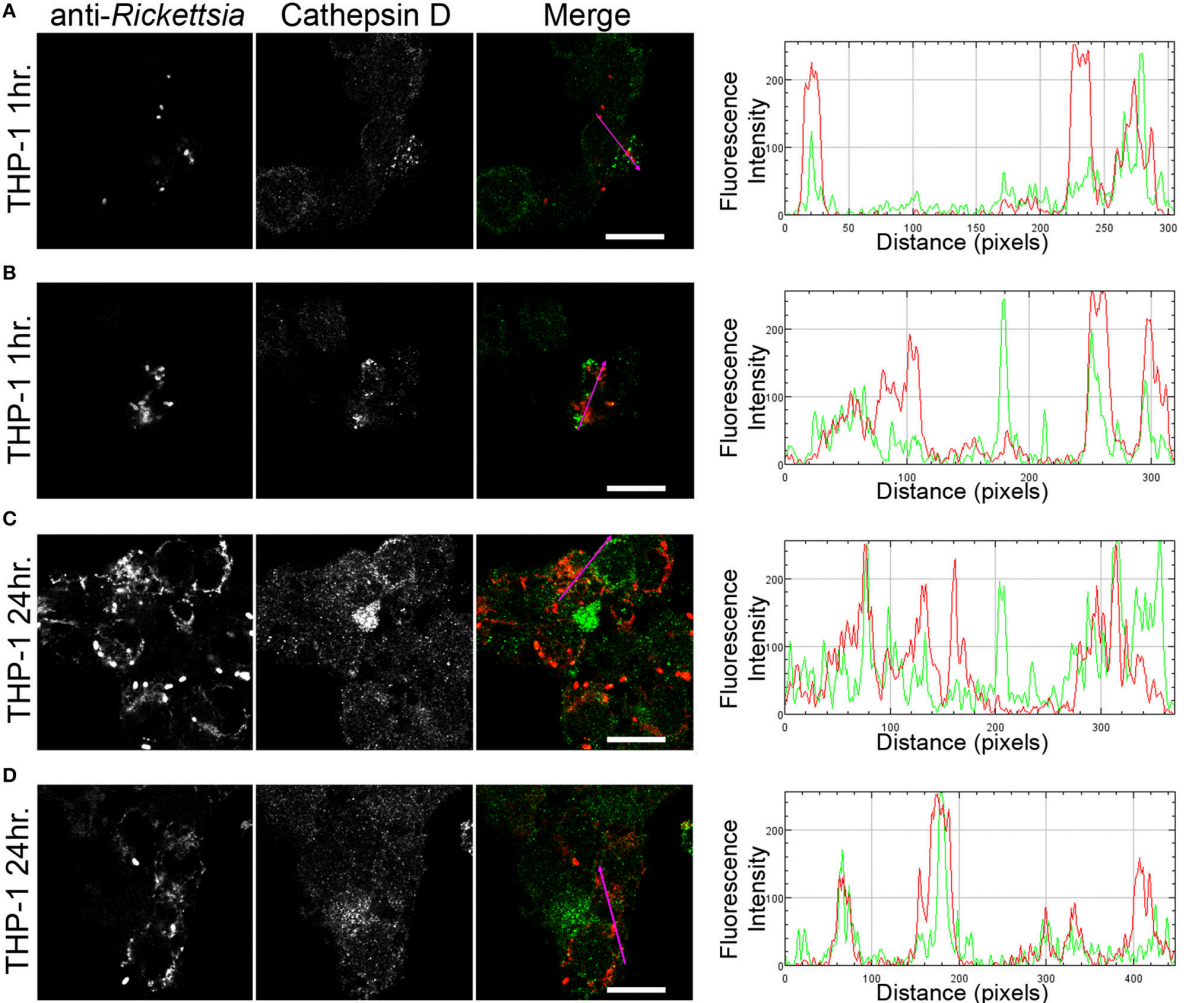
*****R. montanensis*** co-localizes with the lysosomal marker cathepsin D**. THP-1-derived macrophages were infected with *R. montanensis* (MOI = 10). At 60 min or 24 h post infection, cells were fixed, permeabilized, and double stained for immunofluorescence confocal microscopy analysis with NIH/RML I7198 followed by Alexa Fluor 546 (red) to stain *R. montanensis*, and the monoclonal antibody for cathepsin D followed by Alexa Fluor 488 (green). **(A–D)** Representative images of a single slice from the z stacks of THP1-derived macrophages at 60 min post infection **(A,B)** and 24 h post infection **(C,D)**. Each row shows, from left to right, *Rickettsia* staining, cathepsin D staining, the merged image, and a RGB plot profile illustrating the fluorescence intensity along the magenta arrow. Scale bar = 10 μm. Supplementary Movies 9, 10 represent 360° rotation movie of the 3D projection of the stack images shown in **(B)** and **(C)**, respectively.

**Figure 8:**
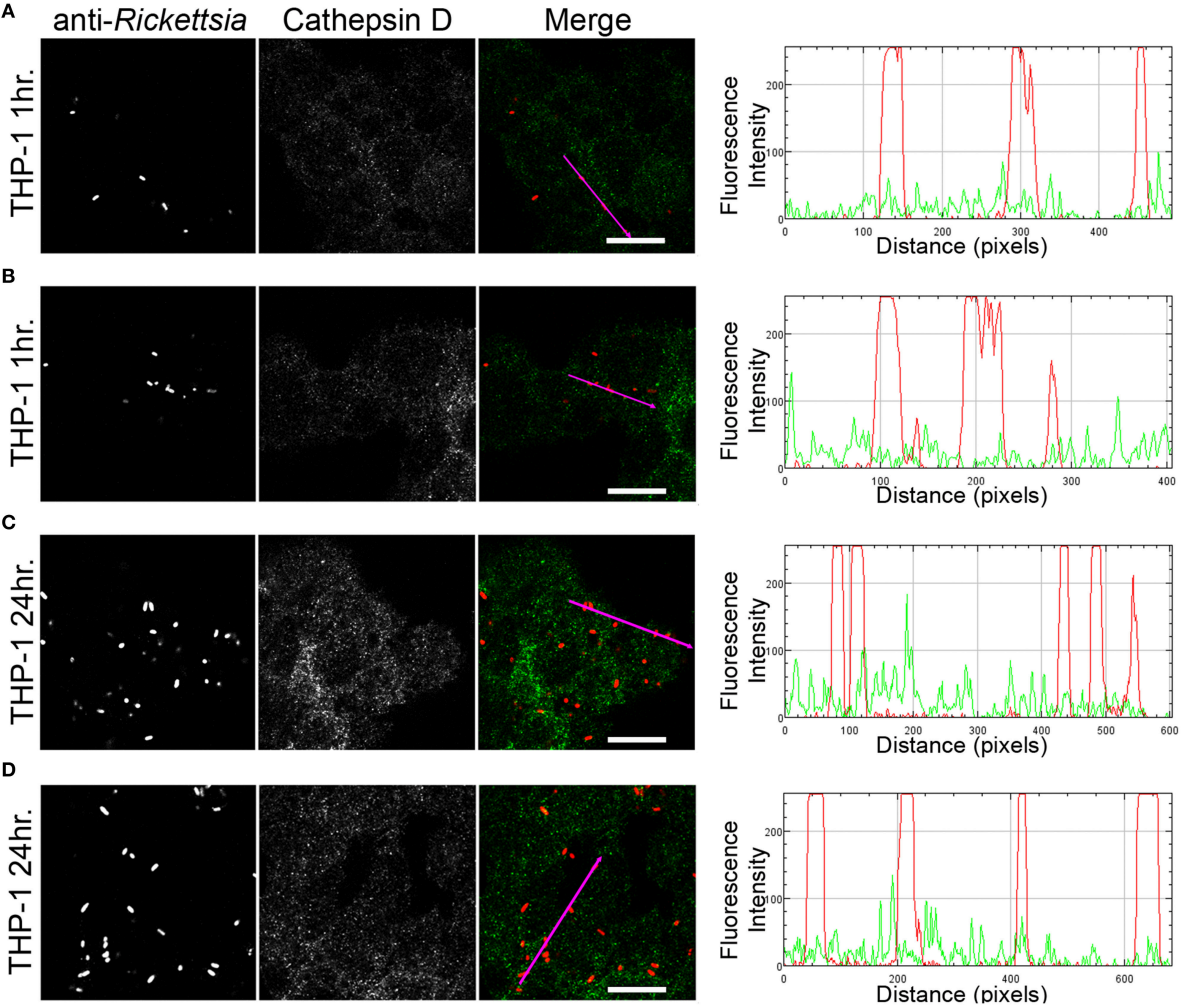
*****R. conorii*** shows no substantial co-localization with the lysosomal marker cathepsin D**. THP-1-derived macrophages were infected with *R. conorii* (MOI = 10). At 60 min or 24 h post infection, cells were fixed, permeabilized, and double stained for immunofluorescence confocal microscopy analysis with anti-Rc_PFA_ followed by Alexa Fluor 546 (red) to stain *R. conorii*, and the monoclonal antibody for cathepsin D followed by Alexa Fluor 488 (green). **(A–D)** Representative images of a single slice from the z stacks of THP1-derived macrophages at 60 min post infection **(A,B)** and 24 h post infection **(C,D)**. Each row shows, from left to right, *Rickettsia* staining, cathepsin D staining, the merged image, and a RGB plot profile illustrating the fluorescence intensity along the magenta arrow. Scale bar = 10 μm. Supplementary Movies 11, 12 represent 360° rotation movie of the 3D projection of the stack images shown in **(B)** and **(D)**, respectively.

We next sought to determine whether the observed lack of *R. montanensis* growth in macrophage-like cells could be attributed to destruction in phagolysosomes. Vero and THP-1-derived macrophages were infected with *R. montanensis* at a MOI of 10 for 1 and 24 h, and then processed for immunofluorescence microscopy using antibodies against rickettsiae and the lysosomal marker, LAMP-2. Again, parallel studies were also performed with *R. conorii* for comparison. Representative slices from z-stack images derived from THP-1 cells at 60 min or 24 h post infection with *R. montanensis* or *R. conorii* are shown in Figures 5, 6, respectively, and those from Vero cells are illustrated in Supplementary Figures 4, 5, respectively. *R. montanensis* in THP-1-derived macrophages at 1 h post-infection do not appear as intact bacteria and at 24 h post-infection, most of the *Rickettsia*-positive staining results from debris that partially localizes to LAMP-2 positive compartments. Analysis of the distribution of fluorescence intensity across selected regions in each panel further shows the substantial overlapping of signals, particularly at 24 h. In contrast, at 1 and 24 h post infection, *R. montanensis* in Vero cells appear intact with very few bacteria co-localizing with LAMP-2 positive compartments (Supplementary Figure 4 and Supplementary Movies 3, 4). As a control, either at 60 min or 24 h post infection in THP-1 or Vero cells, *R. conorii* maintain the morphology of intact bacteria, with no significant co-staining with LAMP-2 positive structures, and proliferate within these two cell types as depicted in an increase in rickettsial cells (Figure 6 and Supplementary Figure 5, Supplementary Movies 7, 8). These observations were further confirmed when infected THP1-derived macrophages were immunostained with an antibody recognizing the mature form of cathepsin D (Kalamida et al., 2014; Lohoefer et al., 2014), one of the most abundant proteases active in the acidic environment of the lumen of lysosomes (Figures 7, 8). *R. montanensis*-positive staining is mostly co-localized with cathepsin D 24 h after infection (Figures 7C,D), and this is further corroborated by the fluorescence intensity profiles showing substantial overlapping between signals. In contrast, no significant co-staining is observed between *R. conorii* and cathepsin D at the same time point, with the representative fluorescence intensity profiles further illustrating very little superposition of signals (Figures 8C,D). Taken together, these results demonstrate a difference in the intracellular fate of *R. montanensis* between epithelial and macrophage cell types and may provide a plausible reason as to why this species is not generally considered a human pathogen.

## Discussion

Differences in pathogenicity and/or virulence between different *Rickettsia* species have been previously reported (Uchiyama, 2012; Wood and Artsob, 2012). Although several genomic, transcriptomic, and proteomic studies between rickettsial species with different levels of virulence have been reported aiming to reveal putative virulence factors, no clear evidence of molecular or biochemical determinants explaining such a dramatic difference were unveiled (Ge et al., 2003; Ellison et al., 2008; Bechah et al., 2010; Clark et al., 2015).

In this work, we evaluated the ability of two SFG rickettsiae with different degrees of pathogenicity in mammals to proliferate within macrophage-like cells. The highly pathogenic, *R. conorii*, and the non-pathogenic *R. montanensis*, were used here as our models of study. Interestingly, the ability of these two SFG rickettsiae to proliferate within THP-1-derived macrophages resulted in a dramatic phenotypic difference. *R. conorii* was found to grow well within macrophage-like cells, and TEM images of THP-1-derived macrophages infected with *R. conorii* at 5 days post-inoculation showed that *R. conorii* is free in the cytoplasm of phagocytic cells, displaying a normal morphology and not surrounded by membranes or phagosome-like structures. On the other hand, the ability of *R. montanensis* to grow within macrophage-like cells was compromised, whereas its ability to grow in either an epithelial (Vero) or endothelial cell line (EA.hy926) was not affected. This phenotype prompted us to evaluate in more detail the known crucial steps of a successful rickettsial infection.

For obligate intracellular bacteria, the concept of a successful *in vitro* infection involves several steps including adherence to a target cell, invasion, avoidance of host defenses and adaptation to the host intracellular environment, multiplication and spread to neighboring cells (Walker and Ismail, 2008). Although the *in vitro* infection process of endothelial and epithelial cells by SFG rickettisae is well studied (Martinez and Cossart, 2004; Martinez et al., 2005), little is known about the molecular details governing the interactions between SFG rickettsiae and professional phagocytes such as macrophages. Our studies of fatal infections in murine models of disseminated disease suggest that the interaction of rickettsiae with cells other than the endothelium during infection may be an underappreciated aspect in rickettsial biology (Riley et al., 2015, 2016). The first step for a successful infection *in vitro* is the binding to or the recognition of the target cell (Bechah et al., 2008a; Walker and Ismail, 2008). Thereby, to start understanding the reason why *R. montanensis* is unable to proliferate in macrophage-like cells, we addressed the adherence capacity of *R. montanensis* to THP-1-derived macrophages and Vero cells. Our results demonstrate that *R. montanensis* is defective in binding THP-1-derived macrophages when compared with their capacity to bind to Vero cells. In contrast, the adherence of *R. conorii* to either epithelial or macrophage-like cells is not affected. Therefore, the difference in the ability of a known human pathogen and a non-pathogenic rickettsial species to bind to macrophage-like cells constitutes a major phenotypic distinction between these two SFG rickettsiae *in vitro*. For endothelial cells, several reports have highlighted the importance of the interactions between rickettsial surface proteins such as the rickettsial surface cell antigens (Sca; Sca0/OmpA, Sca1, Sca2, Sca5/OmpB) with mammalian host cell receptors in mediating adherence and subsequently invasion of cultured mammalian cells (Li and Walker, 1998; Cardwell and Martinez, 2009; Chan et al., 2009, 2010; Riley et al., 2010; Hillman et al., 2013). Amino acid sequence alignments between the rickettsial Sca protein homologs in *R. conorii* and *R. montanensis* reported to play a role in the adhesion to endothelial cells do not reveal any obvious differences sharing between 60.15 and 88.47% of sequence identity (Supplementary Figures 6–9). Nonetheless, we cannot totally rule out that these changes in amino acid sequence may still be responsible for the observed difference in adherence. A gain of function assay, with the noninvasive *E. coli* expressing individual *R. montanensis* Sca proteins, could be a useful tool to assess whether Sca proteins function similarly as has been previously demonstrated (Uchiyama, 2003; Cardwell and Martinez, 2009; Riley et al., 2010). Furthermore, the process by which SFG rickettsiae adhere to macrophage-like cells is not yet studied and we cannot discard the possibility that *R. conorii* and *R. montanensis* may use alternative routes of entry into macrophages. However, the defective ability of *R. montanensis* to bind to THP-1-derived macrophages cannot totally explain the complete lack of growth in macrophage-like cells since rickettsiae can still adhere to these cells.

We demonstrated that the *R. montanensis* cells that are able to adhere to macrophage-like cells still invade these cells. However, the invasion rates of *R. montanensis* appear to be significantly reduced when compared with those obtained for *R. conorii* in both epithelial and macrophage cell lines, further strengthening the possibility that the route by which these two SFG rickettsiae adhere to and invade into macrophage-like cells may indeed be different. Previous reports showed that binding and recruitment of Ku70 to the plasma membrane as well as localized actin rearrangements are important events in the entry of *R. conorii* into non-phagocytic mammalian cells (Martinez et al., 2005; Chan et al., 2009). Furthermore, subsequent studies demonstrated the importance of Sca0/OmpA interactions with α2β1 integrin in the internalization of *R. conorii* into human lung microvascular endothelial cells (Hillman et al., 2013). However, it is unknown if the same events occur upon invasion of *R. conorii* into macrophages. To our knowledge, the mechanism(s) of entry in endothelial and macrophage cells by *R. montanensis* have yet to be elucidated. Therefore, we cannot discard that different SFG rickettsiae can share distinctive mechanism(s) of entry between them. Interestingly, *Legionella pneumophila* strains with different degrees of virulence were shown to differ in their respective mechanisms of entrance into monocytes/macrophages and subsequently in their ability to proliferate within this cell type (Cirillo et al., 1999). Nonetheless, further research is required to better understand the routes of entry in macrophage cells utilized by SFG rickettsiae species of varying degrees of virulence.

We determined that the lack of *R. montanensis* growth in macrophage-like cells also results from the apparent inability of *R. montanensis* to avoid intracellular destruction. Confocal microscopy data demonstrate that intracellular *R. montanensis* are rapidly destroyed in THP-1-derived macrophages, and several bacterial cells co-localized with the lysosomal markers, LAMP-2 and cathepsin D. In contrast, infection of THP-1-derived macrophages by *R. conorii* resulted in no significant co-staining with positive structures for both lysosomal markers and the increase of intact bacteria over the time course of the experiment demonstrate their ability to grow. Interestingly, amino acid sequence alignments of homologous proteins previously reported to mediate rickettsial phagosomal escape, namely membranolytic phospholipase D and haemolysin C (Whitworth et al., 2005), do not demonstrate any obvious difference between *R. conorii* and *R. montanensis* homologs of these two proteins (Supplementary Figures 10, 11). Again, as for Sca proteins, the impact of minor changes in protein sequence and putative protein function cannot be excluded.

Published comparative genomic analysis of the secretome of *R. conorii and R. montanensis* highlight major differences in several genes between these two species, including *rarp*2, encoding Rickettsia Ankyrin Repeat Protein 2 (RARP-2), which is absent in *R. montanensis* genome, and phospholipase A_2_ (Pat-2), which may be present as a pseudogene in *R. conorii* (Gillespie et al., 2015). RARP-2 homologs have been described as virulence factors in other pathogenic bacteria, and *R. typhi* Pat-2 protein was suggested to be necessary to support intracellular survival without affecting host cell integrity (Pan et al., 2008; Rahman et al., 2010, 2013). Whether or not these or other SFG rickettsial gene products contribute to intracellular replication in macrophages needs to be further evaluated.

Together, our results provide supportive evidence that two SFG rickettsiae with different degrees of pathogenicity have opposite fates in macrophage-like cells. Over 40 years ago, Grambrill et al. provided the first evidence that TG rickettsiae strains with different levels of virulence possessed distinct abilities to proliferate in macrophage cell cultures (Gambrill and Wisseman, 1973b). Our results further strengthen the hypothesis that the virulence of different rickettsial species in mammals may somehow be explained by their ability to proliferate within macrophages and potentially other professional phagocytes, and raises the exciting possibility of using macrophage cell cultures as a useful model to predict/understand the pathogenicity of different emerging rickettsial species.

## Author contributions

PC, IS, JM designed experiments and analyzed the data. PC, IS performed the experiments. PC, IS, SR contributed for microscopy immunofluorescence data analysis. PC, IS, JM wrote the paper. All authors discussed the results, interpreted the data, and edited the paper.

## Funding

This work was supported in part by an award to PC (FCT PhD grant SFRH/BD/96769/2013). Research reported in this publication was also supported by the National Institute of Allergy and Infectious Diseases of the National Institutes of Health under Award Number R01AI072606 to JM. The content is solely the responsibility of the authors and does not necessarily represent the official views of the National Institutes of Health.

### Conflict of interest statement

The authors declare that the research was conducted in the absence of any commercial or financial relationships that could be construed as a potential conflict of interest.
