# Supplementary material for: Differences in Intracellular Fate of Two Spotted Fever Group Rickettsia in Macrophage-Like Cells
Source: Front Cell Infect Microbiol. 2016 Jul 29;6:80. doi: 10.3389/fcimb.2016.00080 (PMC4965480; doi:10.3389/fcimb.2016.00080)
Supplement: Supplementary file 13 [file DataSheet1.pdf]

Supplementary Material

Differences in intracellular fate of two spotted fever group *Rickettsia* in macrophage-like cells

Authors: Pedro Curto[1,2,3,5], Isaura Simões[3,4,5], Sean P. Riley[5], Juan J. Martinez[5*]

Affiliations:
[1]PhD Programme in Experimental Biology and Biomedicine, Center for Neuroscience and Cell Biology, University of Coimbra, Portugal;
[2]Institute for Interdisciplinary Research, University of Coimbra, Portugal.
[3]CNC-Center for Neuroscience and Cell Biology, Coimbra, Portugal
[4]Biocant, Biotechnology Innovation Center, Cantanhede, Portugal
[5]Vector Borne Disease Laboratories, Department of Pathobiological Sciences, LSU School of Veterinary Medicine, Baton Rouge, LA, United States

***Corresponding author:** Juan J. Martinez: jmartinez@lsu.edu

Supplementary Movie 1 | THP-1-derived macrophages at 60 minutes post infection with *R. montanensis*
This movie corresponds to Figure 5A and represents a 360 degrees rotation movie of the 3D projection of the stack of images (generated with 3D Viewer, ImageJ). Frame rate, 14 fps. (MOV, 1.35 MB)

Supplementary Movie 2 | THP-1-derived macrophages at 24 hours post infection with *R. montanensis*
This movie corresponds to Figure 5C and represents a 360 degrees rotation movie of the 3D projection of the stack of images (generated with 3D Viewer, ImageJ). Frame rate, 14 fps. (MOV, 1.79 MB)

Supplementary Movie 3 | Vero cells at 60 minutes post infection with *R. montanensis*
This movie corresponds to Supplementary Figure 4A and represents a 360 degrees rotation movie of the 3D projection of the stack of images (generated with 3D Viewer, ImageJ). Frame rate, 14 fps. (MOV, 1.04 MB)

Supplementary Movie 4 | Vero cells at 24 hours post infection with *R. montanensis*
This movie corresponds to Supplementary Figure 4C and represents a 360 degrees rotation movie of the 3D projection of the stack of images (generated with 3D Viewer, ImageJ). Frame rate, 14 fps. (MOV, 739.35 KB)

Supplementary Movie 5 | THP-1-derived macrophages at 60 minutes post infection with *R. conorii*
This movie corresponds to Figure 6A and represents a 360 degrees rotation movie of the 3D projection of the stack of images (generated with 3D Viewer, ImageJ). Frame rate, 14 fps. (MOV, 1.3 MB)

Supplementary Movie 6 | THP-1-derived macrophages at 24hours post infection with *R. conorii*
This movie corresponds to Figure 6C and represents a 360 degrees rotation movie of the 3D projection of the stack of images (generated with 3D Viewer, ImageJ). Frame rate, 14 fps. (MOV, 1.9 MB)

Supplementary Movie 7 | Vero cells at 60 minutes post infection with *R. conorii*
This movie corresponds to Supplementary Figure 5A and represents a 360 degrees rotation movie of the 3D projection of the stack of images (generated with 3D Viewer, ImageJ). Frame rate, 14 fps. (MOV, 747.97 KB)

Supplementary Movie 8 | Vero cells at 24 hours post infection with *R. conorii*
This movie corresponds to Supplementary Figure 5C and represents a 360 degrees rotation movie of the 3D projection of the stack of images (generated with 3D Viewer, ImageJ). Frame rate, 14 fps. (MOV, 2.13 MB)

Supplementary Movie 9 | THP-1-derived macrophages at 60 minutes post infection with *R. montanensis*
This movie corresponds to Figure 7B and represents a 360 degrees rotation movie of the 3D projection of the stack of images (generated with 3D Viewer, ImageJ). Frame rate, 14 fps. (MOV, 1.02 MB)

Supplementary Movie 10 | THP-1-derived macrophages at 24 hours post infection with *R. montanensis*
This movie corresponds to Figure 7C and represents a 360 degrees rotation movie of the 3D projection of the stack of images (generated with 3D Viewer, ImageJ). Frame rate, 14 fps. (MOV, 2.77 MB)

Supplementary Movie 11 | THP-1-derived macrophages at 60 minutes post infection with *R. conorii*
This movie corresponds to Figure 8B and represents a 360 degrees rotation movie of the 3D projection of the stack of images (generated with 3D Viewer, ImageJ). Frame rate, 14 fps. (MOV, 1.98 MB)

Supplementary Movie 12 | THP-1-derived macrophages at 24 hours post infection with *R. conorii*

This movie corresponds to Figure 8D and represents a 360 degrees rotation movie of the 3D projection of the stack of images (generated with 3D Viewer, ImageJ). Frame rate, 14 fps. (MOV, 1.68 MB)

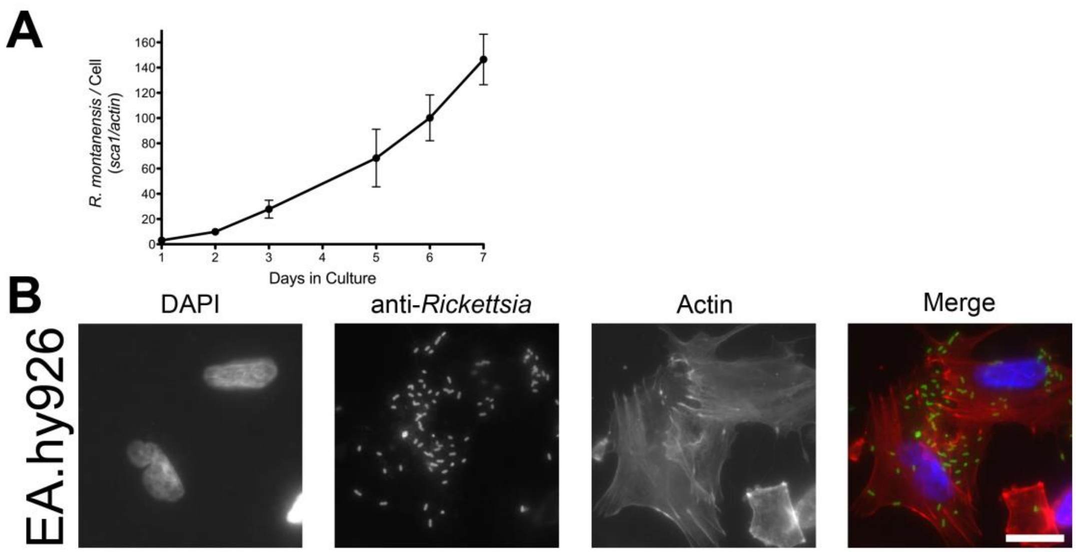

Supplementary Figure 1 | Ability of *R. montanensis* to invade and proliferate within human endothelial cells, EA.hy926. (A) EA.hy926 cells were infected with *R. montanensis* and genomic DNA was extracted at different time-points after infection. Quantitative PCR data are expressed as the ratio of *R. montanensis sca1* versus *actin* DNA content. **(B)** Immunofluorescence microscopy of EA.hy926 cells infected with *R. montanensis* at 3 days post-infection. Cells were stained with DAPI (blue) to identify host nuclei, Phalloidin (red) to stain actin and NIH/RML I7198 followed by Alexa Fluor 488 (green) to stain *R. montanensis*. Scale bar = 10 µm.

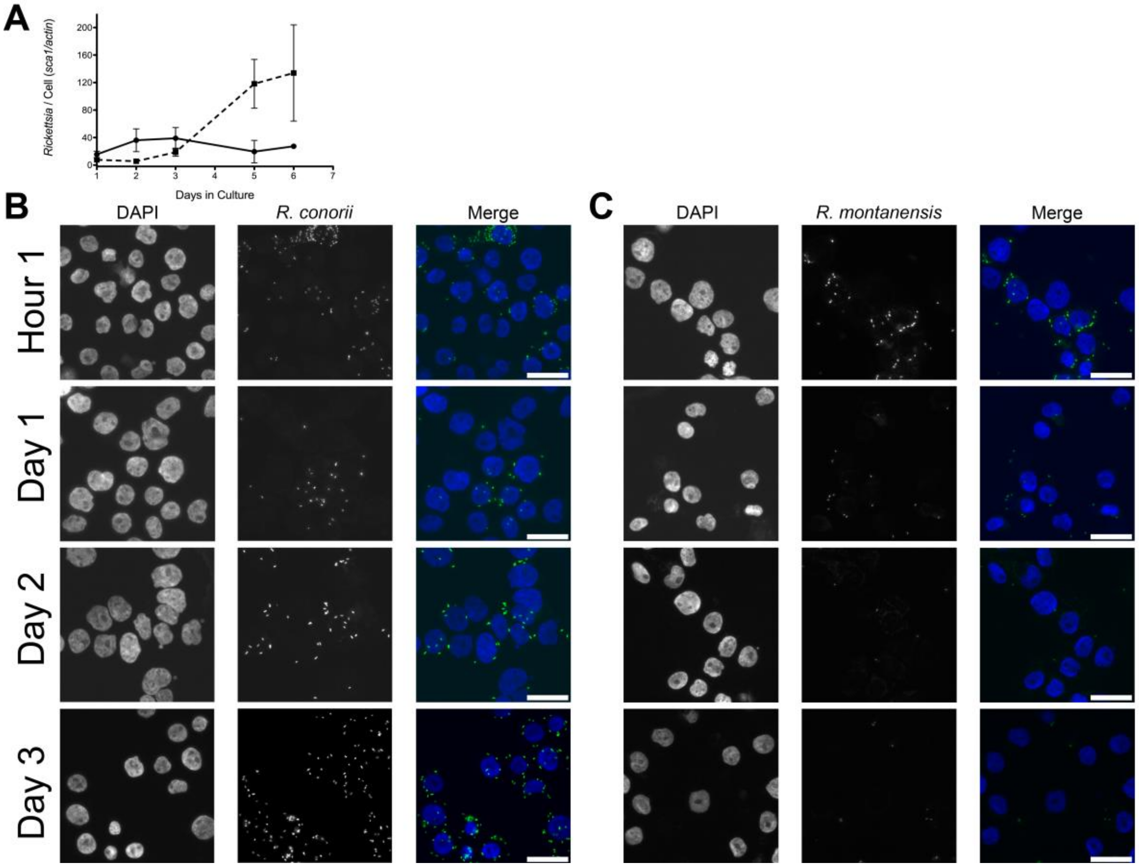

Supplementary Figure 2 | *R. conorii* and *R. montanensis* show a different ability to proliferate within undifferentiated THP-1 cells. (A) Undifferentiated THP-1 cells were infected with *R. montanensis* (solid line) and *R. conorii* (dashed line) and genomic DNA was extracted at different time-points after infection. Quantitative PCR data are expressed as the ratio of *R. montanensis* or *R. conorii sca1* versus *actin* DNA content. **(B)** Immunofluorescence microscopy of undifferentiated THP-1 cells infected with *R. conorii* at 1 h, and 1, 2, and 3 days post-infection, respectively. **(C)** Immunofluorescence microscopy of undifferentiated THP-1 cells infected with *R. montanensis* at 1 h, and 1, 2, and 3 days post-infection, respectively. Cells were stained with DAPI (blue) to identify host nuclei, and anti-Rc_{PFA} or NIH/RML I7198, followed by Alexa Fluor 488 (green) to stain *R. conorii* or *R. montanensis*, respectively. Scale bar = 10 µm.

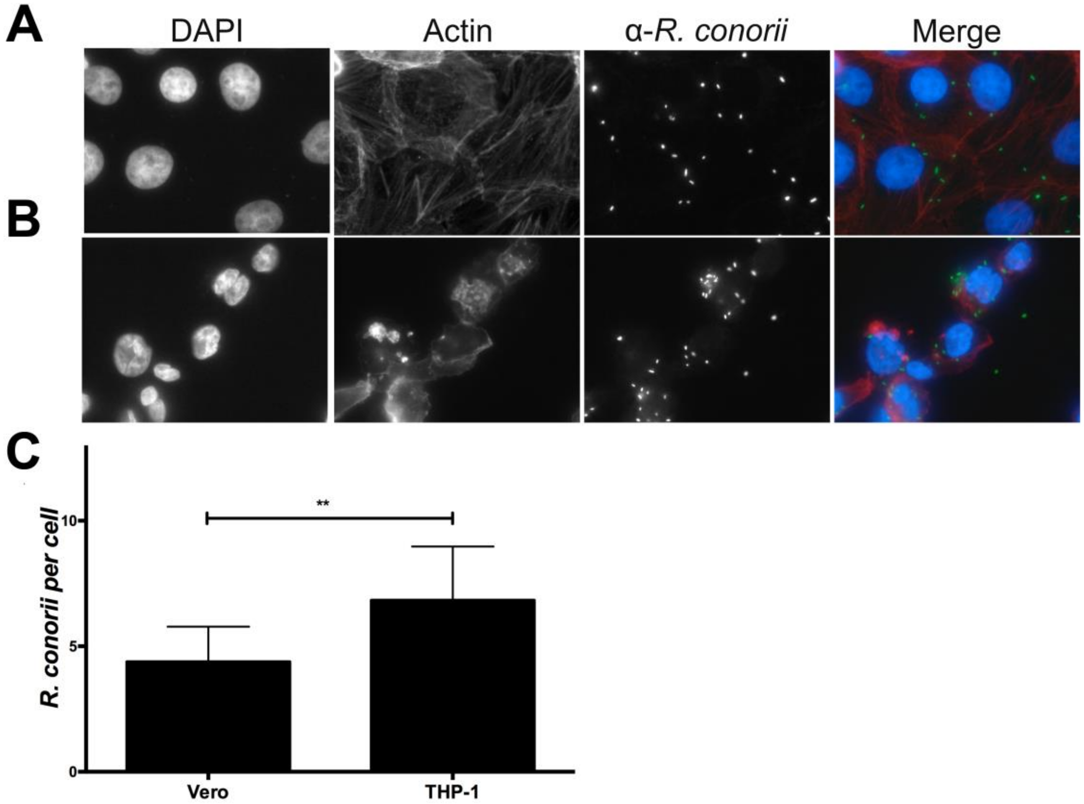

Supplementary Figure 3 | Unlike *R. montanensis*, adherence of *R. conorii* to THP-1-derived macrophages is not decreased. PMA-differentiated THP-1 cells and Vero cells were infected with *R. conorii* (MOI=10). After 60 min of infection, cells were fixed and stained for immunofluorescence analysis with anti-Rc_{PFA} followed by Alexa Fluor 488 (green) to stain *R. conorii*, DAPI to visualize the host nuclei (blue) and Phalloidin to illustrate the host cytoplasm (red). **(A and B)** Representative immunofluorescence images of *R. conorii* association assays in Vero (A) and macrophage-like (B) cells. Each row shows, from the left to right, nuclei staining, actin staining, rickettsia staining and merged images. Scale bar = 10 µm. **(C)** Rickettsia and mammalian cells were counted and results are expressed as the ratio of rickettsia to mammalian cells. At least 200 nuclei were counted for each experimental condition. Results are shown as the mean ± SD (P values: ** <0.01).

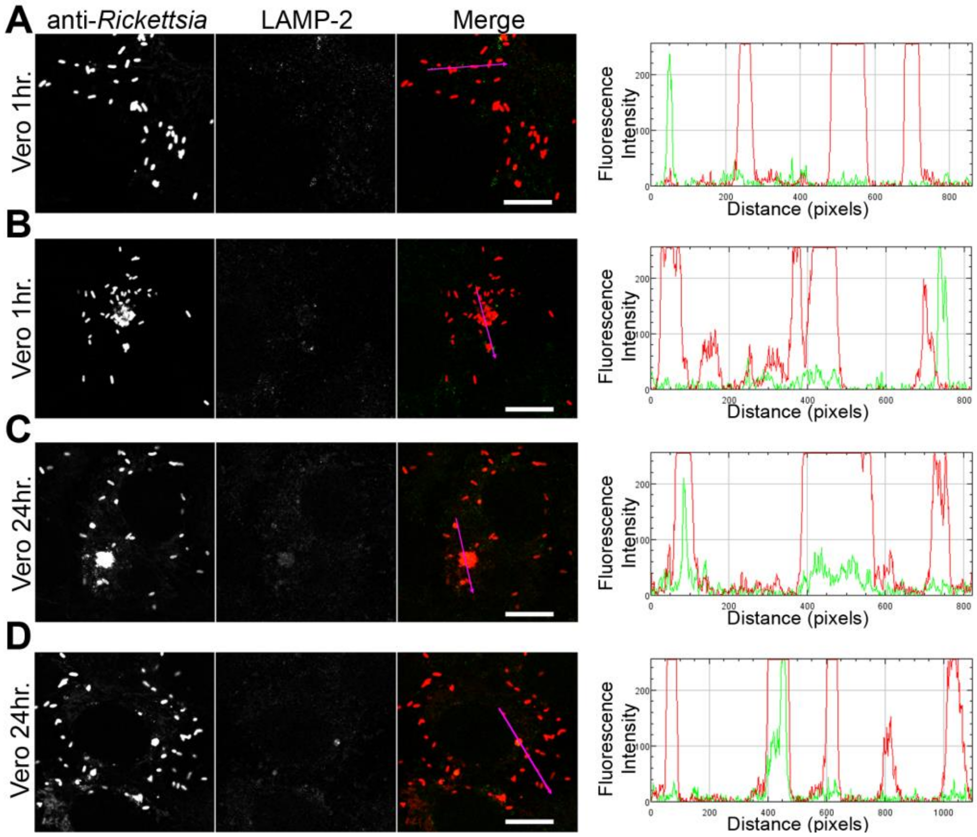

Supplementary Figure 4 | *R. montanensis* is maintained mostly as morphologically intact bacteria in Vero cells. Vero cells were infected with *R. montanensis* (MOI=10). At 60 min or 24 h post infection, cells were fixed, permeabilized and double stained for immunofluorescence confocal microscopy analysis with NIH/RML I7198 followed by Alexa Fluor 546 (red) to stain *R. montanensis* and the monoclonal antibody for LAMP-2, lysosomal membrane protein followed by Alexa Fluor 488 (green). (A-D) Representative images of a single slice from the z stacks. Vero cells at 60 min post infection (A and B) and 24 h post infection (C and D). Each row shows, from left to right, *Rickettsia* staining, LAMP-2 staining, the merged image, and a RGB plot profile illustrating the fluorescence intensity along the magenta arrow. Scale bar = 10 µm. Supplementary movies 3 and 4 represent 360 degrees rotation movie of the 3D projection of the stack images shown in panels 4A and 4C, respectively.

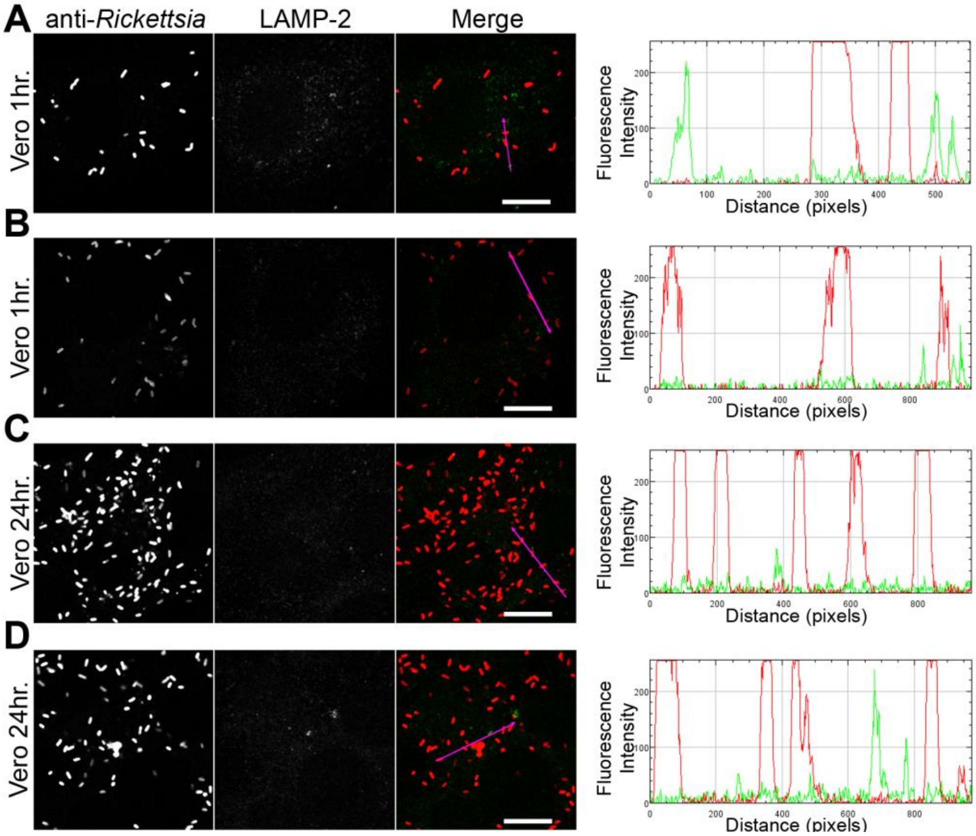

Supplementary Figure 5 | *R. conorii* maintains an intact morphology in Vero cells. Vero cells were infected with *R. conorii* (MOI=10). At 60 min or 24 h post infection, cells were fixed, permeabilized and double stained for immunofluorescence confocal microscopy analysis with anti-Rc$_{PFA}$ followed by Alexa Fluor 546 (red) to stain *R. conorii* and the monoclonal antibody for LAMP-2, lysosomal membrane protein followed by Alexa Fluor 488 (green). (A-D) Representative images of a single slice from the z stacks. Vero cells at 60 min post infection (A and B) and 24 h post infection (C and D). Each row shows, from left to right, *Rickettsia* staining, LAMP-2 staining, the merged image, and a RGB plot profile illustrating the fluorescence intensity along the magenta arrow. Scale bar = 10 µm. Supplementary movies 7 and 8 represent 360 degrees rotation movie of the 3D projection of the stack images shown in panels 5A and 5C, respectively.

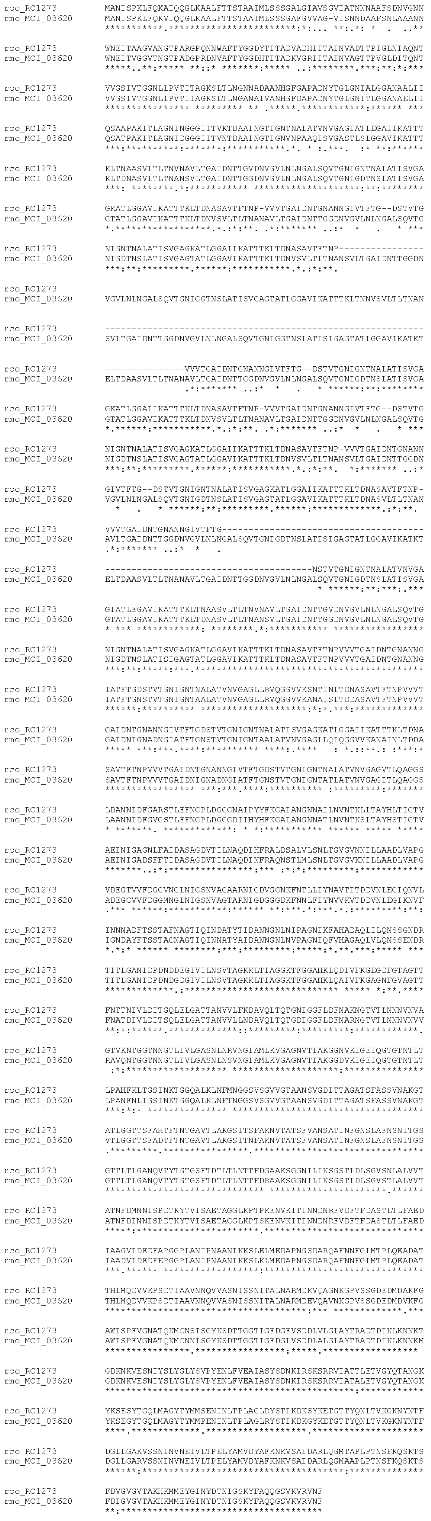

Supplementary Figure 6 | Amino acid sequence alignment of Sca0 protein from *R. conorii* and *R. montanensis.* Amino acid sequences of rickettsial Sca0 from *R. conorii* (RC1273) and *R. montanensis* (MCI_03620) were aligned using the ClustalW software. The alignment resulted in a percentage of sequence identity of 75.36 %.

```
rco_RC0019        MNKLTEQHLLKKSRFLKYSLLASISVG--AAIPFEGMAMSKEAFRIDLSNKLLNHVSQLN
rmo_MCI_04265     MNKLTEQHLLKKSRFLKYSLLASISVGAIIAIPFEGMAMSKEAFCIDLSSKLLNSLSQLN
                  ***************************   ************** ****.**** :****

rco_RC0019        GYKDTTN-----NTPQQIGKVIVSEPKINTYTPSEIREMKISNKPKASNPLKDVPIEDHY
rmo_MCI_04265     GNKDTTNTPDTTNTPQQIGTVIVPEPEINAYIPSEIREMKISNKPKASNPLKDVPVEDHY
                  * *****     *******.***.**:**:* ***********************:****

rco_RC0019        KVVARSKSDVGTARKVRPITRCKTFAGIEKTEQSQNTYTPESTEQMPQKPEIIITASSPT
rmo_MCI_04265     KVVARSKSEVGKARKVRPITRRKTFVGTEKT------------EQMPQKPEIIITASSPT
                  ********:**.********* ***.* ***            *****************

rco_RC0019        VSPASNSFITAPNTPNTTLTSPEHYTTAPGTPSSTPATPYQSTSDSKPNDSLGANTPPNI
rmo_MCI_04265     VSPASNSFVTAPNTPNTTLTSPEHYATAPGTPSSTPVTPYQPTPDSKPNYSLGANTPPNI
                  ********:****************:**********.****.*.***** **********

rco_RC0019        NTNSKAVRRLSFSSSGPQQQAVQSSSQVKSEVPPKPTFVPLPIKKSSTEIVAGMVSNISR
rmo_MCI_04265     NTNSKAARRLSFSSSDPQQQTVQSSSQVKPEVLPESTFVPLPTKKSSTEIAAGVVSNISR
                  ******.********.****:********.** *:.****** *******.**:******

rco_RC0019        VNEMIGIKLAEVTQAIDTTD-KKDKERLQKLYTQLTSTQKTTEKLKSRAEEIETKIKIGE
rmo_MCI_04265     VNEMIGIKLAEVTQAIKDTTGKKNKKPLQKLYTQLTSAQKTTEALKSRVEEIETRIKIGE
                  ****************. *  **:*: **********:***** ****.*****:*****

rco_RC0019        NKDKIKKLEKELTSKNNKADRLFQKIEKIDIPANKVSIKSQETVPVTTASTEVSAFQAQQ
rmo_MCI_04265     NKNKIKKLEKELTSKNNEADRLFQKIEKIDIPTNKVSIKSQETVPVTTASTKVSAFQAQQ
                  **:**************:**************:******************:********

rco_RC0019        ARINEARQGVFNKNKSSGGNARKSSAG-----TKREKKKQEAQKQLSEIKKQEKAIKTAS
rmo_MCI_04265     ARINEAQQVVQNQQYIAAKKTKKQRQAEDKQRAEREKKKQEAQKQLSEIKKQEKAIKTAA
                  ******:* * *::  :. :::*.  .     ::*************************:

rco_RC0019        DKAKEVAASAKKETSRTALRAMQDKMNGDSEQLNKIEENLKLLTPVVYNSSTGPTYKQSP
rmo_MCI_04265     DKAKEVAASAKKETSRTALRAMQDKK---------------LLTPVVYNSSTGSTYKQPP
                  *************************                ************.****.*

rco_RC0019        KATPTIPLSHGVQRILGEQPEDEEGYLVPIKVQQQPYQEIEDP-----------------
rmo_MCI_04265     RAKPTMPLSHRVQRILGEQPEDEEGYLVPMKVQQQPYTVMLPSQEIEDPKSMGSGLGSLS
                  :*.**:**** ******************:*******  :  .

rco_RC0019        --------------------------------------------------------IPSH
rmo_MCI_04265     SGTLGKETDEDVGFYSGWGNMPYTQAANVNSSLEGNLVLGMPQQQIQENRSEVSGQLPSH
                                                                          :***

rco_RC0019        SKDIYEAKVSQYINYLNSIQPNQSNQAQIDSVIDGLATEMRKFSADQFSQKLGEIAHLAS
rmo_MCI_04265     SKDIYEAKASLHINYLNSIQPNQSNQAQIDGVIDSLVAAMRKLSADQFNQKLGDVAHLAS
                  ********.* :******************.***.*.: ***:*****.****::*****

rco_RC0019        IKAYEGLFEKLYEIQQARILETQKVYEQAEISQSYAEYEENSRKSSIPVLSRSSSAKSVI
rmo_MCI_04265     IKAYEGLYSKLYEIQQARILETQKAYEQAEIFQSYAEYEENSRKSGTPVLSRSPSAKSVI
                  *******:.***************.****** *************. ******.******

rco_RC0019        SSNFEEKSALLQTTTTDESLRSDNNWKNSAPYSSSPKLDKRGLEYLDLAGDAFVQNLKQP
rmo_MCI_04265     SSNFEEKSALLQTTTTDESLRSDKNWKNPAPYSSSPKLDK--------------------
                  ***********************:****.***********

rco_RC0019        DTLTIETLGLITPTQNTTVAKSDSSRKNNVSGSISEIQQLQSEKMRTETLGVQDDLGLDL
rmo_MCI_04265     ---------IVTPTQNTTVAKSDSSRKNNASGSIPEIQQLQSEKMRTETLGMQNDLGLDL
                           ::******************.****.****************:*:******

rco_RC0019        HYT-----------------------------------------PQETLTEKSTYLVSSK
rmo_MCI_04265     HSQEERFDSGFRSLESQNLSSAYDFDQLSASLDAAYNQRMNTNAPQETLTEESTYSVSSK
                  *                                           *******:*** ****

rco_RC0019        KKQGNIIKRAVSKVGSILQTNYAENRRRKRDGETSKQRTVDQEGEFGHAWGNENHKESSL
rmo_MCI_04265     KKQGNIIKRAVSKAGSILQTNGAENRKRKRDGETSKQRTVDQEGGFGHAWGNENHKESSL
                  *************.******* ****:***************** ***************

rco_RC0019        SVVSGCIKKATQLISLLDAKRTAILQTTSPSQRRSVSLVLQEIENDYREAIKISQKLQQV
rmo_MCI_04265     SVVSGFTKKATQLISLLDAKRTAILQTTSPSQKKSLNLALQEIENDYQEAIKISQELQQV
                  *****  *************************::*:.*.********:*******:****

rco_RC0019        LIRKPEDIKAYNAKAEKKLDAIKSRADKHFNNIETDVDVGFNPNGGNSHSMPTANMDILP
rmo_MCI_04265     LIRKPEDIKAYNAKAEKKLDAIKSRADKHFNNIETDADVGFNPNWGNSHSMPTANMNNLP
                  ************************************.******* ***********: **

rco_RC0019        KNLAVTPPTNVGSLYNSPQAQQFQEDHKNIIINDRSQG----------------------
rmo_MCI_04265     KNLAIN----VGSLYNSPQAQQFREDHKNIIINAISSISSSGVPKESIDSTITKSLPYLD
                  ****:.    *************:*********  *.

rco_RC0019        -------------------------RLNLVDSTIVRELTDVDASIYNTAPPEVLKEAEAL
rmo_MCI_04265     NDQYQEVVLRVEKVRDINTRGYLLNKLKQLGASEVRELTDVDASIYNIAPPEVLKEAEAL
                                           :*: :.:: ************* ************

rco_RC0019        LDRSQGRLNLVDNTIKKGTQPLSNLSTIYESVSYENLASETIYKTEQPKPSISYTNTSKR
rmo_MCI_04265     LDRSQGRLNLVDSTIKKGTQPLSNLSTIYESVSYENLASETTYKTEQPKPSISYKNTSKR
                  ************.**************************** ************.*****

rco_RC0019        KLPIPLFRSAELDKKLEYLDLEDKLLEVEEARIVKEKEAIAKLNQYQDPENLEFKRLAME
rmo_MCI_04265     KLPIPLLRSAELDKKLDYLDLEDKLLKVEEARIVKEKQAIAKLNQYQDPENLEFKRLAIE
                  ******:*********:*********:**********:********************:*

rco_RC0019        ALDLSSKESQLKQKRKAIEAEFSLNEKSSSTDVSILRSYSIDDISGVLSDAESNLSRSPS
rmo_MCI_04265     ARDLSSKESQLKEKRKAMEAAFSLNKEYSSTDVSMSRSYSIGDISSVPSDAESNLSRSPS
                  * **********:****:** ****:: ******: *****.***.* ************

rco_RC0019        VSGLEDLNNSNVMQLEELKSKHEKIANDYN------------------KELELDTLNKEK
rmo_MCI_04265     VSGLEDLNNSNVMKLGELKSKHEKIANDYKAKELELDTLNHDDPKFKKLKLEVFEILKEK
                  *************:* *************:                   :**:  : ***

rco_RC0019        IWLEGEIKHLDTEFKPKVTESKPVFSCSSSVGSINSFSNDDDLSSRDVVTPVDTLNIEIN
rmo_MCI_04265     IWLEGEIKHLDTESKPKVTESKPVFSCSSSVGSINSLSNGDDLSSR-------DVTIEID
                  ************* **********************:**.******        :.***:

rco_RC0019        KDYVDTYIRVLSNKIQKIEEIQKIEELSGSSSRSEELNHIKEAMAYISNRIQDVEELDEK
rmo_MCI_04265     KDYVDTYIRVLS------NKIQKIEELPGSSSRSEELNHIKEAMAYISNRIQDVEELDEK
                  ************      ::*******.********************************

rco_RC0019        MLLAINAQLQEHDEKISSLLREEAT-----DILAQLLLQEMLVSDGKSESTLPAGDEEQE
rmo_MCI_04265     MLLAINARLQEHDEKISSLLLEKYKGEEAADILAQLLLQDMLASDGKSESTLPAGDKEQE
                  *******:************ *: .     *********:**.*************:***

rco_RC0019        D---------TEVSRQLSSLPALASSNESALALSDDREKECLALGDSSEDEESYDSGFEE
rmo_MCI_04265     DTEDTEDTEVKEVSRQLSSLPALASSNECALALSDDREKECLALGDGSEDEESYDSGFEE
                  *         .*****************.*****************.*************

rco_RC0019        EEETIGQLSDSDGDNLKITEVDTVIPLEQEAKKEMQTQISENAPTLNQAKVVNTIVNNMI
rmo_MCI_04265     EEETIGQLSDSDGGNLKITEVDTAIPLEQEAKKEMQTQISENAPTLNQAKVVNTIVNNMI
                  *************.*********.************************************

rco_RC0019        RNRLDASMNMSNNMVAVGAGDEEESHIKRGLWMRGMYGTNNHGRVENMTGYRGTNKGATI
rmo_MCI_04265     RNRLDASMNMSNNMVAVGAGDEEESHIKRGLWMRGMYGTNNHGRVDNMTGYRGTNKGATI
                  ********************************************:***************

rco_RC0019        GFDAEIDNNIVGIAYSNVHSVFKFKNSKNNDKELINSHVVSIYGQKELPKNFALQALVSA
rmo_MCI_04265     GFDAEIDNNIVGIAYSNVHSVFKFKNSKNNDKELINSHVVSIYGQKELPKNFALQALVSA
                  ************************************************************

rco_RC0019        SKNFIKDKTTYSYGDTKIKSNVKHRNHSYNAEALLHYNYLLQSKLVITPNIGLRYGKSRD
rmo_MCI_04265     SKNFIKDKTTYSYGDTKIRSNVKHRNHSYNVEALLNYNYLLQSKLLITPNIGLRYGKSRD
                  ******************:***********.****:*********:**************

rco_RC0019        GVYNETGVNVQEIALTMKENNILSGIVGTKVTVPLKDALKFNNLGLIFQGAVEHNFKEKT
rmo_MCI_04265     GVYNETGINVQEIALTMKENNILSGIVGTKVTVPLKDALKFNNLGLTFQGAVEHNFKEKT
                  *******:************************************** *************

rco_RC0019        QRINRVVKIFDNTFKHNYLIPKQPKTSYNLGTGIIGSIKNTTISLDYNYYLNKHYRSHQG
rmo_MCI_04265     QRINRVVKIFDNTFKHNYLIPKQPKTSYNLGTGIIGSIKNTTISLDYNYYLNKHYRSHQG
                  ************************************************************

rco_RC0019        SVKLKVNL
rmo_MCI_04265     SVKLKVNL
```

Supplementary Figure 7 | Amino acid sequence alignment of Sca1 protein from *R. conorii* and *R. montanensis.* Amino acid sequences of rickettsial Sca1 from *R. conorii* (RC0019) and *R. montanensis* (MCI_04265) were aligned using the ClustalW software. The alignment resulted in a percentage of sequence identity of 60.15 %.

```
rco_RC0110      MNLQNSHSKKYVLTFFMSTCLLTSSFLSTSARAASFKDLVSKTPAWEKHNSTQQQNIWKD
rmo_MCI_04765   MNLQNSYSKKYVLTFFMSTCLLTSSFLSTSARAASFKDLVSKNPAWEKHNEIQQQNIWKD
                ******:***********************************.*******. ********

rco_RC0110      LTPNEKIKKWQEAALVPSFTQAQNDLGIKYKETDLSSFLDNTRHKARQARAEILLYIERV
rmo_MCI_04765   LTPNEKIKKWQAADLVPSFTQAQDDLGIKYKETDLSSFLDNTRHKARQARAEILLYIERI
                *********** * *********:***********************************:

rco_RC0110      KQQDFDTKKQAYINQGVVPTDIEAATNLGISYDPSKIDNNVEHDQKVRRAEKDKKAVIEL
rmo_MCI_04765   KQQDFDTKKQEYINQGVVPTDIEAATNLGISYDPSKIDNNVENDQKVRRAEKDKKAVIGL
                ********** *******************************:*************** *

rco_RC0110      YVSSINRGIKYKHYVDNDIIPEIQEVRTALNMNKDDAQSFVASIRTEIMENAKGQYIADS
rmo_MCI_04765   YISSINRAIKYKHYVDNDIIPAMKEVRTALNMNKDDAESFVASIRTEIMENAKRQYIADS
                *:*****.************* ::**************:************** ******

rco_RC0110      HIPTEKELKKKFGISRDDNRDGYIKSIRLKVMDKEKPQYIADSHIPTEKELEQKFGADKG
rmo_MCI_04765   HIPTEQEFKNRFGISRDDNRDGYIKSIRLKVMDKEKPQYIAGNSIPTEKELEQKFGADKG
                *****:*:*::******************************.. ****************

rco_RC0110      EATNYIASIATQMMLDKKSYYIDNNIIPNADELMNEFKIGPVKATSYINQIRAGIEANQF
rmo_MCI_04765   EATNYIASIATQMMLGKKSYYIDNNIIPNADELMNEFKIGKIKANSYIDQIRAGMKANQF
                ***************.************************ :**.***:*****::****

rco_RC0110      LNNNDTTKPSTGRSQKKSGSKNDHWYMSNQSINNTGTSARIVTGREKKQRYFFDPISTFK
rmo_MCI_04765   LNNNDTTKPSAGHSQKKSGSKNDNWYMSNQGTNNTGTSSGVSTGRKKKQPYFFDPISTFK
                **********:*:**********:******. ******: : ***:*** **********

rco_RC0110      TYFNTKASKGNLTQSQHNINRIIQQEENIEEFKNLIKTDPIAALTLQVDSSYKQEAVTTI
rmo_MCI_04765   THFNNKESKGNLTQSQRNINRIIQQEENIEEFKNLIKTDPIAALNLEVGSSYKQEAVTTI
                *:**.* *********:***************************.*:*.***********

rco_RC0110      LSDFNDDTIQRVLFSNDKGKLDFNTNIDVKNRPILQELLENSSSEEKTKFAERIQDYATR
rmo_MCI_04765   LSDFNDDTIQRVLFSNDKEQLDFNTNIDVKNRPILKELLENSSSEEKTKFAERIQDYATR
                ****************** :***************:************************

rco_RC0110      NISNSQFEEKARLDLIKLAASKDKSSVENFLTLQLELKNRMQPYVVNSVYILTPEIVKEI
rmo_MCI_04765   NIFNSQFEEKARLDLIKLAASKDKSSVENFLALQLELKNRMQPYIVKSVYILTPEIVKEI
                ** ****************************:************:*:*************

rco_RC0110      NIELKNKGLIRDSLTKDYMIKLAKEVNNHTLNSVIKVILSDSKILSNETNKILGLAVSNN
rmo_MCI_04765   NIELKNKGLIIDSLTKDDMIKLAKAVNKQTLNSVIKVILSDSNMLSNETNKILGLAAGNN
                ********** ****** ****** **::*************::************..**

rco_RC0110      ANNLEQTQSGIPNPPPLPLNGGIPNPPPLPLNGSMPPPP--LHSQGFSSNSKHFDLNQLQ
rmo_MCI_04765   ANNLEQTQSGIPNPPPLPLNGGIPNPPPLPLNGSMPPPPPPLNSQGFSSNSNNFDLNKLQ
                ***************************************  *:********::****:**

rco_RC0110      TEYPHIHSLYVQFTHNTTVQSKAPLQPTASSATSTGRSTPETAYAKLYAEYRTETGGTKA
rmo_MCI_04765   AEYPHIHSLYIQFTR-NTVQPKVPSQPTTSSATSTDRSTPETAYAKLYTEYRTETGGKKA
                :*********:***: .***.*.* ***:******.************:********.**

rco_RC0110      NDLQDQLIKRQADLTNVIRQILTESYANQGADEKTLLNLFSISTPEIAEKAKEAFNTLAQ
rmo_MCI_04765   DDLQDQLIKRQADLTNVIRQILTESYANQGADEKTLVNLFSISTPEIAEKAKEAFNTLVQ
                :***********************************:*********************.*

rco_RC0110      DQYIKDITVNGKKTITSEEIIKNLFNEDTDDAIKRILLSSCKISEELKRPIKLEFNKSEL
rmo_MCI_04765   DPYIQDITVNGKKTITSEEIIKNLFNEDNDDAVKRILLSSCKISEELKKPIKLEFNKSEL
                * **:***********************.***:***************:***********

rco_RC0110      IRELQGKQNPFKQLEFAYINTKNFDQDIFGNRIDELINNPNILTIVQQATFLTKEDTNLR
rmo_MCI_04765   IRELQSKQNPFEQLEFAYTNAKNFDQDIFGNRVKELINNPNILTIGQQATFLITEDTNLR
                *****.*****:****** *:***********:.*********** ****** .******

rco_RC0110      KTINSDQAQAKLDDLRTAILSTIKIEELITANLPQHDFIAIVKEKDPELLKEFLKATTLT
rmo_MCI_04765   KTINSDQAQAKLDDLRTAILSTIKFEELITANLPQHEFIAIVKEKKPELLKEFLKATTIK
                ************************:***********:********.************:.

rco_RC0110      VTGNNNLDQLRLALPSFTGMSNEQIRILSNKLKMSIILKALKECSQEKATQYIHTGNMPP
rmo_MCI_04765   LTGNNNLDQLRLALPSFTDMSNEQVRILASKLNMPIILKALKEHSQEKAKKHIHTGSMPP
                :*****************.*****:***:.**:*.******** *****.::****.***

rco_RC0110      PPPPPPPLPDSQDLELAYLKSLGITKANT--STFKTTPKTYHFSSDIALRYKEFTLSGQK
rmo_MCI_04765   PPPP---LPDSQDLELAYLTSLGITKFNANTSTFKTTPKIYNFSSDIAVRYKEFTLSGQK
                ****   ************.****** *:  ******** *:******:***********

rco_RC0110      SAGYKAKYSDADLLKKAIVESVAFEHSKNLSKAHQNNKYFEQIQKAVNTMYSSFIGHRTE
rmo_MCI_04765   SAGHKAKYSDANLFKKAIVESVAFEHSKNLSKAHQNNKYFAKIQEAVDTMHSSFIGPRTE
                ***:*******:*:************************** :**:**:**:***** ***

rco_RC0110      LEQKIHNIYTSKLLELTKDKEFIKYVEDNIILNKKLTKAFTSADSDFIDSRTELEQKIHN
rmo_MCI_04765   IGQKIHNIYTSKLLELTKDKEFIKYVEDDIILSKKLTEAFTSADSDFIGPRTEIGQKIHN
                : **************************:***.****:**********..***: *****

rco_RC0110      IYIQQLTKYPEEEVKEAFNTASLDFIGPRTEIGQEVHNIYKSQLLELTKDTELCLFTQQV
rmo_MCI_04765   IYTQQLTKYPEEAVKEAFNTANSDFIGPRTEIGQEVHNIYKSQLLELAKDKELFLFVEQL
                ** ********* ********. ************************:**.** **.:*:

rco_RC0110      LAEATELEQKYGSDIQSENSNNEKKVERLDQEKLQLFKQENEATNDESSTKDDTQ-----
rmo_MCI_04765   LVESTELEQKYGSDIQSENSNNEKKIGRLDPKKLRLFQQKNEATNDESSTKDDTQSENSN
                *.*:*********************: *** :**:**:*:***************

rco_RC0110      -----------------------PEDSNKKSEQSDSKTALSPRLLSSNDSKNDKSSDDKK
rmo_MCI_04765   KKSEQSEENEATNDESLTKDDTQPEDSNKKSEQSDSKTALSPRLLSSNDSKNDKSSDDKK
                                       *************************************

rco_RC0110      SLLALRSSDEDDTGYATDEEELEESN-----STTDEELKKDVVLESEDEAIDVSFKTEAI
rmo_MCI_04765   SLLALRSSDEDDKGYETNEEELEESNSTTDEKLTDEELKKDIVLESEDEAIDVSFKTEAI
                ************.** *:********     . ********:******************

rco_RC0110      TEQDEVTQRQQVSDDTSGKVAILVQATSTLHKPVHYNIN-DRLTVAAIGAGDEETSINRG
rmo_MCI_04765   TEQNEATQRQQVSDDTSRKVAILVKATSTLHKPVHYNILSDRLKVAAIGAGDEEASINRG
                ***:*.*********** ******:*************  ***.**********:*****

rco_RC0110      VWISGLYGINKQRIWKNIPKYQNRTTGITIGTDAEFINSHDVIGIAYSRLESQIKYNKKL
rmo_MCI_04765   VWISGLYDINKQGTWKNIPKYQSRTTGITIGTDAEFINSHDVIGIAYSRLESQIKYNKKL
                *******.****  ********.*************************************

rco_RC0110      GKTTVNGHLLSIYSLKELIKGFSLQTITSYGHNYIKNRSKNINNIIGKYQNNSLSFQTLL
rmo_MCI_04765   GKTAVNGHLLSIYGLKELIKGFSLQAITSYGHNYIKNRSKSINNIIGKYQNNNLSFQTLL
                ***:**********.**********:**************.***********.*******

rco_RC0110      NYKYRTKYDLHFIPNIGFQYDYSRASNYKEYNVDIENLMIQKKSNQLFESSLGGKIVFKP
rmo_MCI_04765   NYKYRTKYDLHFIPNIGFKYDYSRASNYKEYNVDIENLLIQKKSNQLFESSLGSKIVFKP
                ******************:*******************:**************.******

rco_RC0110      IVTTNNIVLTPSLYGNIEHHFNNKNTKVNAKATFKGQTLQETIITLKQPKLGYNIGSNIL
rmo_MCI_04765   IVTTNNIVLTPSLYGNIEHHFNNKNTKVNAKATFKGQTLHEKIIIPKQPKLGYNIGSNIL
                ***************************************:*.**  **************

rco_RC0110      MSRKNINVLLEYNYYTHRKYQSHQGLIKLKVNL
rmo_MCI_04765   MSRKNINVLLEYNYYTHRKYQSHQGLIKLKVNL
                *********************************
```

Supplementary Figure 8 | Amino acid sequence alignment of Sca2 protein from *R. conorii* and *R. montanensis*. Amino acid sequences of rickettsial Sca2 from *R. conorii* (RC0110) and *R. montanensis* (MCI_04765) were aligned using the ClustalW software. The alignment resulted in a percentage of sequence identity of 88.47 %.

```
rco_RC1085        MAQKPNFLKKLISAGLVTASTATIVASFAGSAMGAAIQQNRTTNAVATTVDGVGFDQTAV
rmo_MCI_02705     MAQKPNFLKKLISAGLVTASTATIVASFAGSAMGAATQQNRTTNGAATTVDGAGFDQTAA
                  *********************************** *******..******.******.

rco_RC1085        PANVAVPLNAVITAGVNKGITLNTPAGSFNGLFLNTANNLDVTVREDTTLGFITNVVNNA
rmo_MCI_02705     QVNAAVAPNAVITANANNGINFNTPAGSFNGLFLGTANNLAVTVSADTTLGFVTNVANNG
                   .*.**. ******..*:**.:************.***** ***  ******:***.**.

rco_RC1085        NHFNLMLNAGKTLTITGQGITNVQAAATKNANNVVAQVNNGAAIDNNDLQGVGRIDCGAA
rmo_MCI_02705     NSFNLTLGAGKTLTITGQGITNAQAAVTNNAQNVVAQFNGGVAVANNDLSGVGAIDFGAA
                  * *** *.**************.***.*:**:*****.*.*.*: ****.*** ** ***

rco_RC1085        ASTLVFNLANPTTQKAPLILGDNAVIVNGANGTLNVTNGFIKVSSKSFATVNVINIGDGQ
rmo_MCI_02705     ASTLVFNLAEPTTQKAPLILGGNAVIANGVNGTLNVTNGFIQVSDKSFATVKAINIGDGQ
                  *********:***********.****.**.***********:**.******:.*******

rco_RC1085        GIMFNTDADN-VNTLNLQANGATITFNGTDGTGRLVLLSKNAAATDFNVTGSLGGNLKGI
rmo_MCI_02705     GFIFNTNVAAGGNALNLQVGGATINFNGTDGTGRLVLLSKNGAATDFNVTGSLGGNLKGI
                  *::***:.    *:****..****.****************.******************

rco_RC1085        IEFNTVAVNGQLKANAGANAAVIGTNNGAGRAAGFVVSVDNGKVATIDGQVYAKDMVIQS
rmo_MCI_02705     IEFNTVAVAGQLIANAGPANAVIGTNNDAGRAAGFVVSVGNGNAATITGQVYAKDMVIQS
                  ******** *** ****.  *******.***********.**:.*** ************

rco_RC1085        ANAVGQVNFRHIVDVGTDGTTAFKTAASKVAITQNSNFGTTDFGNLAAQIIVPNTMTLNG
rmo_MCI_02705     ANAGGQVNFGHIVDVGTDGTTAFKTAASKVAITQNSNFGTTDFGNLAAQITVPDTMTLTG
                  *** ***** ****************************************** **:****.*

rco_RC1085        NFTGDASNPGNTAGVITFDANGTLASASADANVAVTNNITAIEASGAGVVQLSGTHAAEL
rmo_MCI_02705     NFTGNASNPENTAGVITFAANGTLASASADANVAVTNNITAIEASGVGVVQLSGTHTAEL
                  ****:**** ******** ***************************.*********:***

rco_RC1085        RLGNAGSVFKLADGTVINGKVNQTALVGGALAAGTITLDGSATITGDIGNAGGAAALQGI
rmo_MCI_02705     RLGNAGSVFKLADGTVINGKVNQTAVVGGALAAGAITLDGSATITGDIGNGGGAAALQGI
                  *************************:********:***************.*********

rco_RC1085        TLANDATKTLTLGGANIIGANGGTINFQANGGTIKLTSTQNNIVVDFDLAIATDQTGVVD
rmo_MCI_02705     TLANDATKTLTLGGANIIGANGGTINFQANGGTIKLTSTQNNILVDFDLAINTDQTGVVD
                  *******************************************:******* ********


rco_RC1085        ASSLTNAQTLTINGKIGTVGANNKTLGQFNIGSSKTVLSDGDVAINELVIGNNGAVQFAH
rmo_MCI_02705     ASSLTNAQTLTISGTIGTVGANNKTLGQFNVGSSKTALNGGNVAINELVIGNNGSVQFAH
                  ************.*.***************:*****.*..*:*************:*****

rco_RC1085        NTYLITRTTNAAGQGKIIFNPVVNNNTTLATGTNLGSATNPLAEINFGSKGAANVDTVLN
rmo_MCI_02705     DTYLITKTTNAAGQGKIIFNPVVNNNTTLAAGTNLGSAANPLAEINFGSK-GAHADTILN
                  :*****:***********************:*******:*********** .*:.**:**

rco_RC1085        VGKGVNLYATNITTTDANVGSFIFNAGGTNIVSGTVGGQQGNKFNTVALDNGTTVKFLGN
rmo_MCI_02705     VGKGVNLYATNITTTDANVGSFVFNAGGTNIVSGTVGGQQGSKFNTVELDNGTTVKFLGN
                  **********************:******************.***** ************

rco_RC1085        ATFNGNTTIAANSTLQIGGNYTADFVASADGTGIVEFVNTGPITVTLNKQAAPVNALKQI
rmo_MCI_02705     AMFNGGTKIEAKSTLQIGGNYTADFVASADGTGIVEFVNTGPITVTLNKQAAPVGVLKQM
                  * ***.*.* *:******************************************..***:

rco_RC1085        TVSGPGNVVINEIGNAGNYHGAVTDTIAFENSSLGAVVFLPRGIPFNDAGNRIPLTIKST
rmo_MCI_02705     TVSGPGNVVVNEIGNAGNAHGAVTGTIAFENSSLGATLFLPSGIPFNDVT------IKST
                  *********:******** *****.***********.:*** ******.       ****

rco_RC1085        VGNKTATGFDVPSVIVLGVDSVIADGQVIGDQNNIVGLGLGSDNDIIVNATTLYAGIGTI
rmo_MCI_02705     VGNETAKGFDAPKVIVSGVDSVIADGQVIGDQNNIVGLGLGSDNGIIVNATKLYAGIGTT
                  ***:**.***.*.*** ***************************.******.*******

rco_RC1085        NNNQGTVTLSGGIPNTPGTVYGLGTGIGASKFKQVTFTTDYNNLGNIIATNATINDGVTV
rmo_MCI_02705     NNNQGTVILSGGVPNTPGTVYGLGKDASASQLKQVTFTTDYNNLGNIIATNTTINDGVTV
                  ******* ****:***********.. .**::*******************:********

rco_RC1085        TTGGIAGIGFDGKITLGSVNGNGNVRFVDGILSHSTSMIGTTKANNGTVTYLGNAFVGNI
rmo_MCI_02705     TTGGIAGTVFNGKITLGSVNGNANVRFADGILSNSTSMIVTTKANNGTVTYLGGAYVGSI
                  *******  *:***********.****.*****:***** *************.*:**.*

rco_RC1085        GDSDTPVASVRFTGS----DGGAGLQGNIYSQVIDFGTYNLGISNSNVILGGGTTAINGK
rmo_MCI_02705     GASNTPVASVRFTGSGTGSDNGAVLQGNIYSQVIDFGTYDLDIVNSNVILGGGTTAINGA
                  * *:***********    *.** ***************:*.* ***************

rco_RC1085        INLRTNTLTFASGTSTWGNNTSIETTLTLANGNIGNIVILEGAQVNATTTGTTTIKVQDN
rmo_MCI_02705     IDLCTNTLTFASGTSTLGNNTSIETTLTLADGNIGHIVIAEGARVNATTTGTTTINVQDK
                  *:* ************ *************:*****:*** ***:***********:***:

rco_RC1085        ANANFSGTQTYTLIQGGARFNGTLGGPNFVVTGSNRFVNYGLIRAANQDYVITRTNNAEN
rmo_MCI_02705     ANANFSGTQTYTLIQGGARFNGTLGDPDFAVTGSNRFVNYGLIRAANQDYVITRTNNVEN
                  *************************.*:*.***************************.**

rco_RC1085        VVTNDIANSSFGGAPGVGQNVTTFVNATNTAAYNNLLLAKNSANSANFVGAIVTDTSAAI
rmo_MCI_02705     VVTNDIASSTFGGALGVGQNVTTFVNATNTAAYNNLLLAKNSADSANFVGSIVTDTSAAV
                  *******.*:**** ***************************:******:********:

rco_RC1085        TNAQLDVAKDIQAQLGNRLGALRYLGTPETAEMAGPEAGAIPAAVAAGDEAVDNVAYGIW
rmo_MCI_02705     TNVQLDVAKNIQAQLGNRLGALRYLGTPETAEMAGPEAGAIPAAVAAGDEAVDNVAYGIW
                  **.******:**************************************************

rco_RC1085        AKPFYTDAHQSKKGGLAGYKAKTTGVVIGLDTLANDNLMIGAAIGITKTDIKHQDYKKGD
rmo_MCI_02705     AKPFYTDAHQSKKGGLAGYKAKTTGVVIGLDTLANDNLMIGAAIGITKTDIKHQDYKKGD
                  ************************************************************

rco_RC1085        KTDVNGFSFSLYGAQQLVKNFFAQGSAIFSLNQVKNKSQRYFFDANGNMSKQIAAGHYDN
rmo_MCI_02705     KTDVNGFSFSLYGAQQLVENFFAQGSAIFSLNQVKNKSQRYFFDANGNMSKQIAAGNYDN
                  ******************:*************************************:***

rco_RC1085        MTFGGNLTVGYDYNAMQGVLVTPMAGLSYLKSSDENYKETGTTVANKQVNSKFSDRTDLI
rmo_MCI_02705     MTFGGNLTVGYDYNAMQGVLVTPMAGLSYLKSSDENYKETGTTVANKQVNSKFSDRTDLI
                  ************************************************************

rco_RC1085        VGAKVAGSTMNITDLAVYPEVHAFVVHKVTGRLSKTQSVLDGQVTPCISQPDRTAKTSYN
rmo_MCI_02705     VGAKVAGGTMNITDLVVYPEAHAFVVHKVNGRLSKTQSVLDGQVTPCISQSDRTAKTSYN
                  *******.*******.****.********.********************.*********

rco_RC1085        LGLSASIRSDAKMEYGIGYDAQISSKYTAHQGTLKVRVNF
rmo_MCI_02705     LGLSASIRPDAKMEYGIGYDAQIASKYTAHQGTLKVRVNF
                  ********.**************:****************
```

Supplementary Figure 9 | Amino acid sequence alignment of Sca5 protein from *R. conorii* and *R. montanensis*. Amino acid sequences of rickettsial Sca5 from *R. conorii* (RC0019) and *R. montanensis* (MCI_02705) were aligned using the ClustalW software. The alignment resulted in a percentage of sequence identity of 88.14 %.

```
rco_RC1270          MKRKNNKFIEISIAFILGIALGLYGQNPDYFTNLISQKSLALSALQIKHYNISELSRSKV
rmo_MCI_03585       MKRRNNKFIEISTAFILGIALGIYGQNPDYFTNLISQKSLALPALQIKHYNISELSRSKV
                    ***:********* *********:******************.*****************

rco_RC1270          STCFTPPAGCTKFIANQIDKAEESIYMQAYGMSDALITTALINAQARGVKVRILLDRSNL
rmo_MCI_03585       STCFTPPAGCTKFIANQIDKAEESIYMQAYGMSDELITTALINAQARGVKVRILLDRSNL
                    ********************************** *************************

rco_RC1270          KQKFSKLHELQRAKIDVDIDKVPGIAHNKVIIIDKKKVITGSFNFTAAADKRNAENVIII
rmo_MCI_03585       KQKFSKLHELQQAKIDVGIDKVPGIAHNKVIIIDKKKVITGSFNFTAAADKRNAENVIII
                    ***********:*****.******************************************

rco_RC1270          EDQELAESYLQNWLNRKASN
rmo_MCI_03585       EDQELAESYLQNWLNRKASN
                    ********************
```

Supplementary Figure 10 | Amino acid sequence alignment of membranolytic phospholipase D protein from *R. conorii* and *R. montanensis*. Amino acid sequences of rickettsial phospholipase D from *R. conorii* (RC1270) and *R. montanensis* (MCI_03585) were aligned using the ClustalW software. The alignment resulted in a percentage of sequence identity of 96.50 %.

```
rmo_MCI_02955      MLKSSKKEDSSKKNQNNKLIFTVQKLFSPIKNFFRKTKTPDNFFGVIKRLKINSQKMTLD
rco_RC1141         MLKSSKKEDSSKKNQNNKLIFTVRKLFSPIKNFFRKTKTPDNFFGVIKRLKINSQKMTLD
                   ***********************:************************************

rmo_MCI_02955      ERNILANLLELEDKTIEDIMVPRSDIAAIKLTTNLEELSESIKLEVPHTRTLIYDGTLDN
rco_RC1141         ERNILANLLELEDKTIEDIMVPRSDIAAIKLTTNLEELSESIKLEVPHTRTLIYDGTLDN
                   ************************************************************

rmo_MCI_02955      VVGFIHIKDLFKALATKQNGRLKKLIRKHIIAAPSMKLLDLLAKMRRERTHIAIVVDEYG
rco_RC1141         VVGFIHIKDLFKALATKQNGRLKKLIRKHIIAAPSMKLLDLLAKMRRERTHIAIVVDEYG
                   ************************************************************

rmo_MCI_02955      GTDGLVTIEDLIEEIVGRIDDEHDQQLDSDNFKVINNSTIISNARVEVEVLEEIIGEKLQ
rco_RC1141         GTDGLVTIEDLIEEIVGRIDDEHDQQLDSDNFKVINNSTIIANARVEVEVLEEIIGEKLH
                   *****************************************:*****************:

rmo_MCI_02955      NDDDEFDTIGGLVLTRVSSVPAIGTRIDISENIEIEVTDATPRSLKQVKIRLKNGLNGK
rco_RC1141         NDYDEFDTIGGLVLTRVSSVPAIGTRIDISENIEIEVTDATPRSLKQVKIRLKNGLNGQ
                   ** *******************************************************:
```

Supplementary Figure 11 | Amino acid sequence alignment of haemolysin C protein from *R. conorii* and *R. montanensis*. Amino acid sequences of rickettsial haemolysin C from *R. conorii* (RC1141) and *R. montanensis* (MCI_02955) were aligned using the ClustalW software. The alignment resulted in a percentage of sequence identity of 98.34 %.

Supplementary Table 1 | Primers and probes used in q-PCR assays.

Primer name	Primer sequence	Concentration of primer or probe in PCR reaction
**Actin-F420**	5’-CCTGTATGCCTCTGGTCGTA-3’	300 nM
**Actin-R681**	5’-CCATCTCCTGCTCGAAGTCT-3’	300 nM
**Actin-Hex**	5’-/5MAXN/ACTGTGCCC/ZEN/ATCTAC-3’	200 nM
**Sca1-F5271**	5’-CAAGCTCGTTATTACCCCGAAT-3’	300 nM
**Sca1-R5371**	5’-CTACCGCTCCTTGGAATGTTAGACC -3’	300 nM
**Sca1-Fam**	5’-/56-FAM/TCGGCTTAA/ZEN/GATACGGGAAGT-3’	200 nM
